# Unveiling the distribution and research patterns of *Aspergillus* spp. in Saudi Arabia: a systematic and bibliometric analysis

**DOI:** 10.3389/fmicb.2025.1638271

**Published:** 2025-08-19

**Authors:** Kholoud K. Alzahrani

**Affiliations:** Department of Biology, University College of Umluj, University of Tabuk, Umluj/Tabuk, Saudi Arabia

**Keywords:** *Aspergillus*, Saudi Arabia, bibliometric analysis, ecological distribution, institutional research, international collaboration

## Abstract

*Aspergillus* species play an important role in agriculture and human health, however their detection, distribution and research in Saudi Arabia have not yet been fully described. For this reason, the objective of this study was to review the progress, places where *Aspergillus* research is being carried out and its long-range strategies in Saudi Arabia over the last 54 years. Applying advanced bibliometric processes, we analyzed a 520 scientific articles recorded a 108 different *Aspergillus* species from 275 diverse environments. Research productivity demonstrated distinct evolutionary phases, progressing from limited output (1970–1980) through steady development (1990–2000) to remarkable acceleration during 2010–2018 (peaking at 41 publications in 2013), followed by stabilization at 11–22 publications annually. Institutionally, 33 Saudi universities contributed, with King Saud University leading (172 publications, 39%). International collaboration networks showed strong Egypt-Saudi partnerships complemented by linking with India, Australia, and the USA. The research detected main five species received significant research focus: *A. niger* (421 studies), *A. flavus* (297), *A. fumigatus* (204), *A. terreus* (174), and *A. ochraceus* (105), reflecting strategic prioritization of agricultural, industrial, and clinical significance. Samples of isolates were taken from a variety of locations, as soil (114 studies and encompassing 8 major subcategories), plants (184 sources) and food (32 sources) are the most common types. By analyzing strategic thematic mapping, it was found that the area successfully moved away from mainly medical issues toward a mix that includes agricultural, environmental and biotechnological matters. Details of multiple *Aspergillus* species in various Saudi Arabian habitats are important additions to global science and help meet local needs in farming, healthcare and industry. The results prove that concentrating efforts and developing institutions can enhance research and solve local problems.

## 1 Introduction

*Aspergillus* is one of the most common and ecologically genera of filamentous fungi, the impact of it is significant within the fields of human health, agriculture, and environmental studies more broadly (Bennett, 2007; Abdel-Azeem et al., 2019). These are very versatile organisms, able to exist in microenvironments ranging from dry deserts to hospitals (Schmiedel and Zimmerli, 2016). Nonetheless *Aspergillus* species have increasingly attracted the attention of researchers in Saudi Arabia in the past few decades due to their role as clinically relevant opportunistic infections and as a key ecological component in the unique environment of the Arabian Peninsula (Khateb et al., 2025). Despite growing interest in these subjects, our understanding remains limited concerning their dissemination, the total number of extant species, or the nature of the research conducted on them. This underscores the imperative for a comprehensive literature review.

The Arabian Peninsula, encompassing deserts, coastlines, and expanding urban regions, presents diverse ecological habitats for fungi. Approximately 80% of the peninsula’s land area lies within Saudi Arabia’s borders (Almazroui et al., 2012), and across its varied ecological zones, *Aspergillus* species have consistently demonstrated high adaptability and prevalence (Alotaibi et al., 2020). Empirical research has demonstrated that *Aspergillus* is the most prevalent fungal genus across deserts, agricultural lands, and indoor environments (Khateb and Alkhaibari, 2023; Sajer et al., 2024). This extensive distribution underscores its environmental significance and the need for an in-depth understanding of its dissemination and morphological variations. The convergence of arid climatic conditions, sandstorms, and evolving healthcare infrastructures has engendered unique mycological phenomena in Saudi Arabia. Alotaibi et al. (2020) reported that in AlQasab and Taif, *Aspergillus* spp. constituted the predominant fungi in desert soils, accounting for 34.5% of the fungal isolates in these areas. These fungi exhibit a preference for arid environments and utilize sudden dust storms to disseminate spores, which are implicated in respiratory infections (Sajer et al., 2024). Deserts serve as reservoirs for *Aspergillus* species capable of enduring harsh conditions (Khateb and Alkhaibari, 2023). Globally, research has primarily concentrated on *Aspergillus fumigatus*-related diseases; however, *A. flavus*, *A. niger*, and *A. terreus* are more frequently isolated in clinical samples within Saudi Arabia (Rudramurthy et al., 2019; Kmeid et al., 2020; Khateb et al., 2025). An estimated 2.7% of Saudi patients with asthma have been diagnosed with allergic bronchopulmonary aspergillosis (ABPA), with studies revealing a predominance of *A. niger* over *A. fumigatus*, which deviates from the global norm as delineated by AlShareef (2020). Consequently, it is imperative to conduct region-specific studies to gain a deeper understanding of localized ecological and clinical dynamics.

Although *Aspergillus* is a significant cause of numerous infections, the capacity for diagnosing fungal diseases is inconsistent across medical centers in Saudi Arabia. Recent research drawing data from 57 hospitals indicates that a mere 32% of these institutions performed examinations or analyses of mycological samples (Khateb and Alkhaibari, 2023). Laboratories predominantly rely on traditional culture and microscopy methods, which allow their personnel to identify fungi solely at the genus level. It is infrequent for clinics to conduct outdated antifungal susceptibility testing, serological assays, and molecular diagnostics; thus, results are often processed by external laboratories and may sometimes be reported incompletely. The utilization of *A. fumigatus* tests may fail to detect conditions induced by other clinically relevant *Aspergillus* species such as ABPA. Deficiencies in diagnostic tools imply that the prompt and precise detection of invasive fungal diseases in the region is hindered by the current mycological infrastructure (Khateb et al., 2024). Environmental surveys reveal that *Aspergillus* species are prevalent in numerous locations throughout Saudi Arabia. *Aspergillus* ranks as the second most prevalent fungal pathogen in Saudi hospitals, accounting for 17% of all fungal infections according to recent studies (Khateb and Alkhaibari, 2023). Moreover, *Trichoderma* remains the predominant fungal genus in desert soils, and isolates of this genus exhibit strong resistance to extreme temperatures and produce beneficial biochemicals (Ameen et al., 2022). Such adaptability is essential for both the environment and human health. From an alternative perspective, researchers have begun to concentrate on the potential that native *Aspergillus* strains hold for biotechnology. As reported by Sajer et al. (2024), both *A. flavus* and *A. niger* isolated from Sabkha marshes synthesize bioactive compounds with potent antibacterial and anticancer properties. Desert-isolated organisms actively produce a range of enzymes, including cellulases, laccases, lipases, proteases, amylases, and chitinases (Ameen et al., 2022). These findings reveal the untapped industrial and therapeutic potential of Saudi fungal biodiversity.

Bibliometric analysis quantifies publication trends, structures, and impacts (Salinas-Ríos, 2022; Kinawy et al., 2024). They have improved how research results are shared and analyzed. Tracking and analyzing research ensures better quality in science and promotes equal opportunities to use scientific advances for all (Boichenko and Zinchenko, 2022). Analyses of these indicators help us better understand the numbers and significance of scientific output in different fields according to Vinkler (2010).

This study seeks to address this gap by conducting a comprehensive analysis of research pertaining to *Aspergillus* species within the context of Saudi Arabia. The study examines the sequence of publications, delineates the primary domains of inquiry, identifies the collaborative networks of scientists, and assesses the attribution of scholarly credit. Furthermore, the research maps the distribution of *Aspergillus* species across various Saudi regions, recognizes significant species utilized in scientific research. The findings elucidate the current status of *Aspergillus* research in Saudi Arabia, underscore areas necessitating further investigation, and propose future research pathways to support effective health, agricultural, and environmental management.

## 2 Methodology

**FIGURE 1:**
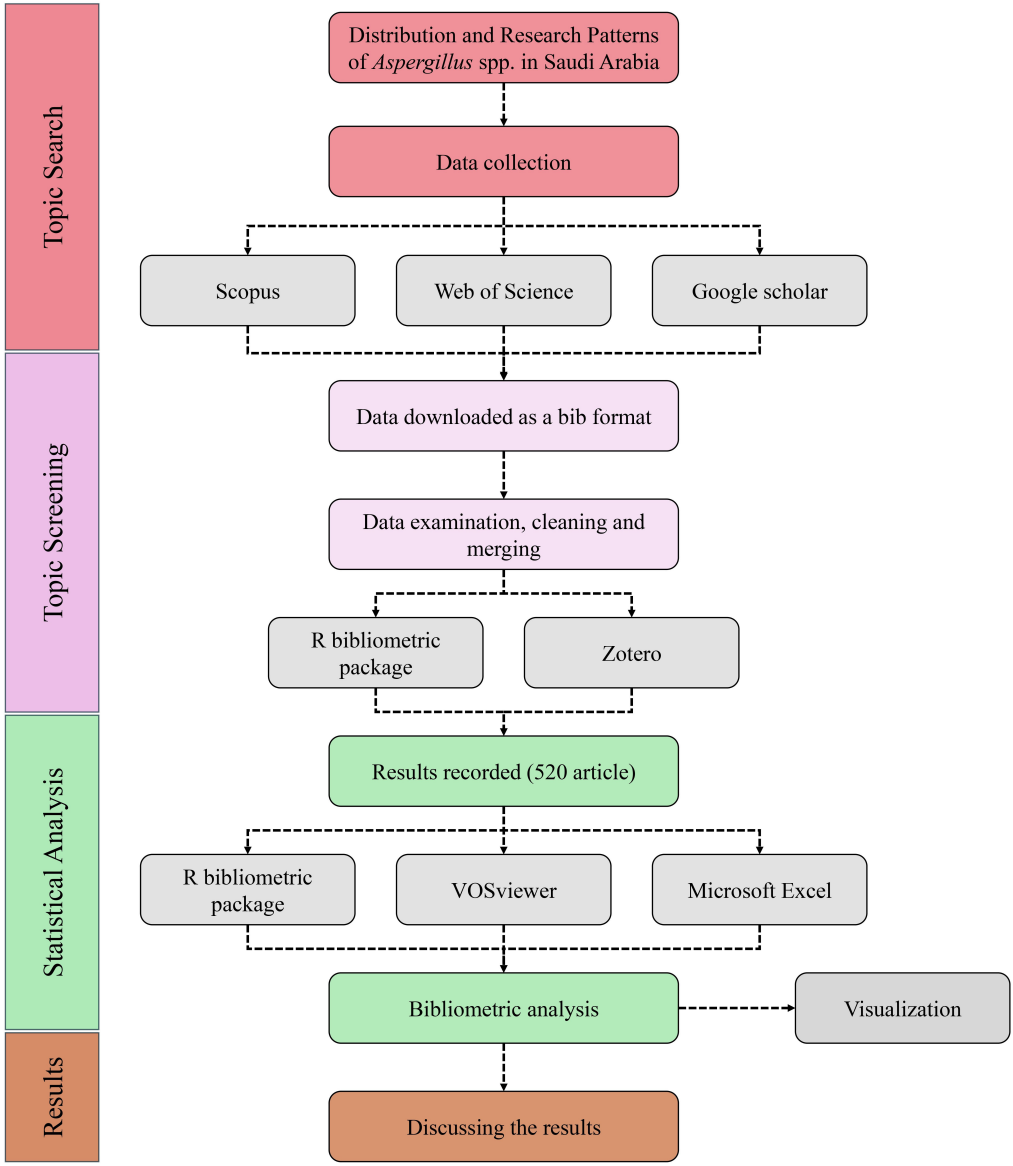
Flowchart shows the bibliometric methods utilized in this investigation. The procedure comprises four stages: (1) Topic Identification, (2) Topic Evaluation, (3) Statistical Examination, and (4) Results Analysis.

A meticulous bibliometric methodology was employed to examine the studies on *Aspergillus* species within Saudi Arabia. The secondary data was explored and analyzed through four distinct yet interrelated procedures: identifying the topic, conducting a screening process, performing statistical analysis, and interpreting the findings, as illustrated in (Figure 1). Utilizing this method, information from articles was systematically collected, organized, and examined. In the initial phase of the research, literature searches were conducted using three academic databases: Scopus, Web of Science, and Google Scholar. The search strings used included: [“*Aspergillus*” AND “Saudi Arabia” AND (“distribution” OR “prevalence” OR “isolates”)]. Boolean operators (AND/OR) were employed to refine results and ensure comprehensive coverage. Searches were limited to articles published in English from 2000 to 2024, and document types were restricted to journal articles and reviews to ensure quality and relevance. Consistent search criteria across all platforms were implemented to mitigate bias and facilitate more straightforward comparisons of materials. A multi-database strategy was imperative to ensure the inclusion of any content indexed exclusively on a single platform. All the gathered data was converted into (.bib) format using BibTeX in the second step to keep bibliographic references accurate for later stages. All references were brought into Zotero and R bibliometrix to clean it, remove duplicates and standardize the data. According to set standards, only articles including information on *Aspergillus* species found in Saudi Arabian areas were reviewed. As a result of a complete review, 520 articles were picked for careful bibliometric analysis. At the last stage, collected and cleaned data files were further analyzed using statistics and networks. The bibliometrix package (R-Development-Core-Team, 2021) available in RStudio 2025.05.0 + 496 version was utilized to examine how many publications were made, how collaborative the authors were and trends in publications. For this research, VOSviewer (van Eck and Waltman, 2019) was used to make visual representations of most isolated strains, sources, keyword co-occurrence and bibliographic coupling networks. Additional descriptive statistics were made possible through Microsoft Excel, GraphPad prism and Origin. This detailed analysis allowed the findings on publication volumes, how research spreads in Saudi regions, cooperation among institutions and how research topics evolved to be detected. A combination of numbers and network analysis with insights from experts was used to find main research issues, gaps in knowledge and developing trends. Special attention was given to the distinct features of *Aspergillus* research in Saudi Arabia, including work done by institutions, difficulties in making diagnoses and its importance in the environment.

### 2.1 Data collection and processing

For our subjects, we accessed and reviewed publications from all suitable sources, giving additional preference to the Scopus database, then Clarivate Analytics Web of Science (WoS) database and finally Google Scholar. To make certain our findings are accurate and free from false positives, we looked for studies using a topic filter based on roles, covering the title, abstract, keyword plus and author keywords. In addition, we carefully examined the collected articles to ensure their relevance to our subjects and isolated *Aspergillus* spp. from Saudi Arabia. As shown in Table 1, 520 articles were approved, ranking for a growth of 5.24% every year. The average number of citations per document was 7.35 and were written by 1002 different researchers. More than 88 of the total works were conducted by single authors and among the chosen works, 3.48% involved a collaboration with international contributors.

**TABLE 1:** Main data features of the collected research of *Aspergillus* species in KSA.

Description	Results
**Main information about data**	
Timespan	1971:2024
Documents	520
Annual growth rate %	5.24
Document average age	16
Total citations	3821
Average citations per doc	7.35
**Total production overview**	
Saudi universities	33
Other Saudi organizations affiliation	25
Foreign universities	75
Other foreign organizations affiliation	10
Saudi journals	17
Foreign journals	266
**Document contents**	
Keywords plus (ID)	2660
Author’s keywords (DE)	852
**Authors**	
Authors	1002
Authors of single-authored docs	88
**Authors collaboration**	
Single-authored docs	147
Co-authors per doc	3.08
International co-authorships %	3.48
**Documents**	
Articles in Saudi journals	66
Articles in foreign journals	454
Total studies in these articles	729
Isolated *Aspergillus* species	108
Isolation main sources	10
Total different isolation sources	275

### 2.2 Network analysis methods

Bibliometric network analysis serves as a comprehensive tool enabling the examination and investigation of the relationships and behaviors inherent in scientific articles (Karimi et al., 2021). Networks, as mentioned by Knoke and Yang (2019), comprise a set of actors or nodes connected via various types of interactions termed edges within Social Network Analysis (SNA). As bibliometric data continues to expand, the interconnections between bibliographic units evolve into comprehensive networks capable of revealing latent developments and emergent trends within specific research domains. The utilization of this analytical methodology facilitates theoretical advancement and elucidates pathways for further academic inquiry, as noted by Khan and Wood (2016). In this study, we employed social network analysis methodologies to examine bibliometric data, concentrating on the number of participants involved, the percentage of all potential connections, and the distance between the two most distant participants. The integration of these measures facilitates an evaluation of the structure and robustness of the academic network. A crucial component of bibliometric analysis involves the examination of scientific collaboration networks from the perspectives of researchers, institutional groups, or nations. Significant advancements and enhanced comprehension primarily arise from collaborative team efforts. Zou et al. (2018) established that articles authored by multiple contributors tend to achieve greater success in being published in prominent journals and garnering citations. Furthermore, the manner in which institutions collaborate plays a decisive role in shaping the advancement of various scientific fields and informs the formulation of policies (Ding, 2011). It’s also possible to see the framework of a subject by examining the network of related terms. Studying how important terms are combined gives researchers an idea of the main topics, common ideas and new areas emerging in various texts (Radhakrishnan et al., 2017).

### 2.3 Thematic analysis framework

Through theme mapping or strategic diagramming, we make the evolution of themes in a research domain clearer. Previously, it was established by Law et al. (1988) and then refined by Callon et al. (1991) by integrating density and centrality metrics to determine the significance and developmental stage of each thematic cluster. These maps present outcomes derived from both co-word analysis and portfolio analysis, effectively delineating the evolution of research themes over time (Zong et al., 2013). To enhance the functionality of theme maps, conceptual structure maps dissect a research domain into discrete knowledge clusters while elucidating the significant interconnections among them. This approach proves instrumental in unearthing novel insights and identifying interdisciplinary connections among studies within the literature. Monitoring scholarly output over time constitutes a crucial component of bibliometric research. A key method involves detecting citation bursts, which manifest when a substantial number of articles simultaneously reference analogous papers, thereby indicating burgeoning and promising topics within an academic field (Kim and Chen, 2015). To attain a more comprehensive perspective, reliance was placed on Google Scholar citations, owing to their greater abundance compared to those identified in Scopus and WOS. Further, co-citation analysis underscores publications that are frequently cited concurrently, potentially because they address analogous subjects or processes. Conversely, bibliographic coupling connects articles utilizing identical sources, thereby unveiling topics being jointly examined by disparate research groups. The presence of numerous cross-references between materials or authors suggests substantial thematic similarity. In essence, keyword co-occurrence analysis aids in constructing thematic frameworks and identifying emerging trends. By cataloging the joint occurrences of terms, researchers can discern the significance of topics and observe the evolving interests within specific fields over time. Embedded within every well-structured plan is an understanding of individual keyword usage. The examination of co-authorship and the social networks of scholars and institutions elucidates their interconnectedness. The findings articulate the contributions, the collaborative nature of scientists, and identify the most active research centers. Ullah et al. (2022) argue that co-authorship analysis reveals the interactions among scientists and across research fields.

## 3 Results

### 3.1 Bibliometric overview and trends

Research publications on *Aspergillus* species from Saudi Arabia spanned five decades (1971–2024) and feature 520 scientific articles as shown before in Table 1. According to the literature, there has been an increasing interest in this field, with a stable yearly rate of 5.24%. The average age of the documents is 16 years which indicates this is a well-established field that still gathers interest. Overall, the documents have received 3,821 citations and with an average of 7.35 per document, this suggests that they have received moderate attention and notice in the scientific world. Beside the research scene involved a diverse institutional participation, with 33 Saudi universities and other 25 Saudi organizations contributing to the research base. Additionally international cooperation is recognizable from the diverse group of 75 international universities and 10 global partner organizations involved. It is further clear that publication distribution has a strong liking for international dissemination, with 454 articles published in 266 foreign journals compared to only 66 articles in 17 local Saudi journals. This 87.3% predominance of foreign journal publications suggests researchers prioritize international visibility and impact for their findings, potentially seeking wider readership and higher citation potential. The intellectual structure of the research is represented by 2,660 Keywords Plus and 852 author-provided keywords. This substantial keyword volume indicates a diverse range of topics and approaches within *Aspergillus* research in Saudi Arabia. The disparity between system-generated and author-provided keywords may reflect the multifaceted nature of the research themes beyond what authors explicitly identify. Authorship patterns showed that there are 1,002 authors in this field and 88 of them released single-authored works. Based on the metrics, 147 documents (or 28.3%) were written by a single person, and most documents were completed collaboratively, with an average of 3.08 contributors to each document. This type of collaboration means scientists are involved both alone and as part of a group investigation. Although international institutions are very active (75 universities), the low co-author rate suggests that institutions are more involved than individual foreign researchers. The data presented 729 investigations published in 520 papers, indicating that many papers cover several experiments. Over 108 *Aspergillus* species were identified from 10 main isolation sources and in total, 275 verified samples were used. The thorough work done here clearly represents the wide-ranging ecological diversity of *Aspergillus* in several Saudi Arabian locations. By choosing international platforms, scientists aim to bring together scientific communities worldwide. Even though 7.35 citations per document are good for this research, there is still space for improvement by finding new partners and directing efforts. Only 3.48% of the studies here had international co-authors, even though almost 20% came from foreign institutions. This points to a way to increase true collaboration among international researchers.

**FIGURE 2:**
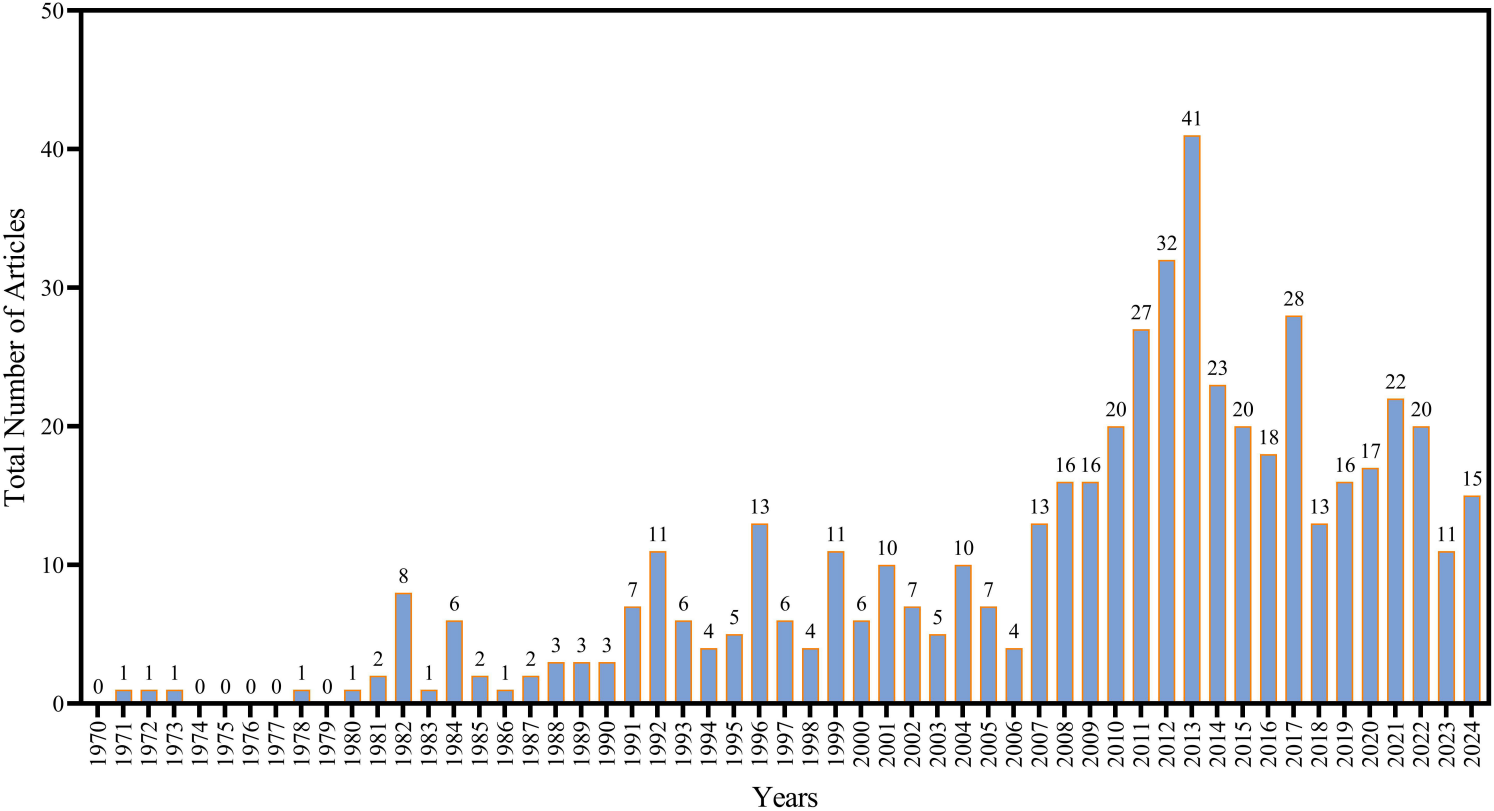
Annual scientific production of *Aspergillus* species research in Saudi Arabia.

An investigation of the chronological distribution of publications on *Aspergillus* in Saudi Arabia revealed a clear phase of research advancement (Figure 2). A primary phase (pre-1990), scientific output was negligible and unpredictable. Whereas from 1970 to 1979, purely four publications were recognized. This constrained pattern persisted until the early 1980s, with annual yields being low until a little rise was noted in 1982, which recorded eight publications. While between 1983 and 1989, the quantity of publications remained constant at 1–2 pieces year. Whereas the establishment phase from 1990 to 2005 indicates the initiation of a gradual rise in research output. Throughout this timeframe, yearly throughput varied between 3 and 13 publications. Significant peaks were observed in 1992 (11 publications), 1996 (13 publications), 1999 (11 publications), and 2001 (10 publications). This stage shows the development of sustained research attention in *Aspergillus*, demonstrating an increasing acknowledgment of its significance within the Saudi scientific community. During the growth phase (2006–2012), a clear expansion was observed. Throughout these years, the annual research output consistently increased from 13 articles in 2007 to 32 in 2012, effectively duplicating research productivity. Further the focus on the peak production phase (2013–2018) represents the zenith of research activity. In 2013, the output reached a peak with 41 publications. Other years with considerable productivity include 2014 (23 publications), 2015 (20 publications), and 2016 (18 publications). This growth is aligned with the overall advancement of Saudi Arabia’s research infrastructure and global academic collaborations during the same period. In the Recent Stabilization Phase (2019–2024), research output has stabilized, ranging between 11 and 22 publications per year. This maintenance reveals the field’s maturation, characterized by consistency, although somewhat reduced, productivity relative to the peak years. The current phase may suggest a transition toward the merging of existing knowledge and a focus on research quality and specialization.

The 20 most important publication sources for *Aspergillus* species study in Saudi Arabia show different patterns of distribution and thematic priority (Table 2). These journals published 162 papers, which is around 31.2% of the total 520 documents assessed. The Journal of Pure and Applied Microbiology is the most common place to publish, with 28 articles (5.4%) published there. This fits well with the microbiological and applied nature of a lot of *Aspergillus* research. After that, the Saudi Journal of Biological Sciences has 20 articles (3.8%), which shows how important national publications are for spreading information in the region. Mycopathologia comes in third with 16 articles (3.1%), showing that there is a lot of interest in fungal pathology and its effects on health. The places where the publications are held show a strong worldwide focus. The Saudi Journal of Biological Sciences, the Journal of King Saud University, and the Annals of Saudi Medicine are the only three of the top 20 journals that are located in Saudi Arabia. Together, they publish 30 articles. There are 17 additional publications in regional journals, such as the Pakistan Journal of Biological Sciences (9 articles), the Pakistan Journal of Botany (3 articles), and the Arab Gulf Journal of Scientific Research (5 articles). This shows that scientists in the region are working together. The African Journal of Microbiology Research (14 articles) and the African Journal of Biotechnology (13 articles) together publish 27 papers, which shows that there are connections between Africa and other continents. The Journal of Laryngology & Otology, Journal of Food Protection, and PLOS ONE are examples of Western and international journals that show how Saudi *Aspergillus* research is spreading over the world. The choice of journals also shows some of the main study themes. There is a medical focus in articles published in periodicals like the Saudi Medical Journal, The Journal of Laryngology & Otology, and Annals of Saudi Medicine that talk about the clinical and pathological aspects of *Aspergillus* infections. Food safety is another important issue, and journals like the Journal of Food Protection and Foodborne Pathogens and Disease publish research on contamination caused by aflatoxins. The Fresenius Environmental Bulletin talks about issues that affect the environment. The presence of PLOS ONE, however small, implies that more people are becoming interested in open access and open research methods. Saudi scholars seem to be following a diverse publication strategy. The prominence of outputs in specialized microbiological journals, constituting approximately 17% of the top sources, indicates a deliberate endeavor to engage expert audiences. The representation of Saudi-based journals, accounting for 18.5%, suggests continued support for national publishing platforms. However, the limited presence of articles in high-impact international journals indicates an opportunity to exert a greater impact and increase citation frequency.

**TABLE 2:** Most 20 relevant sources for the most published articles about scientific production of *Aspergillus* species isolated from Saudi Arabia.

Journal name	Total articles
Journal of Pure and Applied Microbiology	28
Saudi Journal of Biological Sciences	20
Mycopathologia	16
African Journal of Microbiology Research	14
African Journal of Biotechnology	13
Pakistan Journal of Biological Sciences	9
Saudi Medical Journal	6
The Journal of Laryngology & Otology	6
Journal of King Saud University - Science	6
Journal of Food Protection	5
Genetics and Molecular Research	5
Arab Gulf Journal of Scientific Research	5
Foodborne Pathogens and Disease	5
Fresenius Environmental Bulletin	4
Annals of Saudi Medicine	4
Digest Journal of Nanomaterials and Biostructures	4
Pakistan Journal of Botany	3
PLOS ONE	3
Assiut Veterinary Medical Journal	3
Australian Journal of Basic and Applied Sciences	3

On the other side, (Table 3) presents the 20 most frequently cited articles on *Aspergillus* species. These highly cited papers together account for 1,488 citations recorded across all 520 documents in the comprehensive analysis. The highly cited group spans three decades (1991–2020), with notable variations in publication date and citation accumulation. Early pioneering works from 1991 to 1999 create 30% of the top cited papers, establishing foundational knowledge in the field. While mid-period studies from 2000 to 2010 acquired another 30% of highly cited papers, demonstrating a sustained research excellence during this establishment phase. After that recent research from 2011 to 2020 reports for 40% of the most cited publications, implying increasing quality and relevance of contemporary Saudi *Aspergillus* research, which associates with the previously observed publication peak during 2013–2018. This distribution indicates both the enduring value of early work and the growing recognition of more recent contributions. It reveals that citation metrics and varying patterns of academic impact across this temporal spectrum. The most highly cited article of Alghamdi et al. (2014) has accumulated 138 citations, however the 20th ranked paper Al-Garni et al. (2009) has collected 50 citations, demonstrating a relatively gradual rather than precipitous decline in citation counts throughout this elite group. While recent papers display higher annual citation rates, as Ali et al. (2020) leading at an impressive 12.67 citations per year, followed by Alghamdi et al. (2014) at 11.50, and Alsaggaf et al. (2020) at 10. This pattern reveals deeper contemporary attraction in recent publications despite their shorter exposure time, suggesting accelerating recognition of Saudi Arabian contributions. Whilst considering for time of publication through normalized citation scores, Alghamdi et al. (2014) holds the highest impact with a score of 10.45, followed by Al-Hindi et al. (2011) at 7.99 and Ouf et al. (2015) at 7.59, implying these studies have precise significance within the field relative to their publication age.

**TABLE 3:** Highest cited articles according to Scopus and WOS, about scientific production of *Aspergillus* species isolated from Saudi Arabia.

Paper	Total citations	TC per year	Normalized TC
ALGHAMDI M, 2014, SCI TOTAL ENVIRON	138.00	11.50	10.45
OUF S, 2015, J SCI FOOD AGRIC	105.00	9.55	7.59
HASHEM M, 2010, SAUDI J. Biol. Sci.	103.00	3.17	3.92
ELLIS M, 1994, EUR J CLIN MICROBIOL INFECT DIS	98.00	3.06	4.76
AMEEN F, 2016, SAUDI J BIOL SCI	95.00	9.50	6.40
CAMERON J, 1991, ARCH OPHTHALMOL	84.00	2.40	6.72
ELGORBAN A, 2016, MYCOSPHERE	78.00	7.80	5.26
ALI K, 2020, PROCESS BIOCHEM	76.00	12.67	4.47
KHAIRALLAH S, 1992, DOC OPHTHALMOL	74.00	2.18	3.22
TABBARA K, 1998, OPHTHALMOLOGY	70.00	2.50	3.84
ALRAJHI A, 2001, AM J TROP MED HYG	67.00	2.68	4.50
AL-HINDI R, 2011, AFR J MICROBIOL RES	63.00	4.20	7.99
ALSAGGAF M, 2020, ADV POLYM TECHNOL	60.00	10.00	3.53
KAMESWARAN M, 1992, J LARYNGOL OTOL	56.00	1.65	2.43
ABU E A, 1999, EUR J OPHTHALMOL	56.00	2.07	5.45
AL-OTHMAN M, 2014, DIG J NANOMAT BIOSTR	55.00	4.58	4.16
BU R, 2005, J MED MICROBIOL	55.00	2.62	3.93
SALEEM A, 2014, J TAIBAH UNIV SCI	53.00	4.42	4.01
GONDAL M, 2012, J ENVIRON SCI HEALTH PART A TOXIC HAZARD SUBST ENVIRON ENG	52.00	3.71	5.84
AL-GARNI S, 2009, AFR J BIOTECHNOL	50.00	2.94	4.17

Medical mycology signifies a noteworthy focus, with eight papers (40%) appearing in journals dedicated to ophthalmology, respiratory medicine, tropical medicine, and clinical microbiology. Which contain highly cited article about aspergillosis (Cameron et al., 1991; Khairallah et al., 1992), also respiratory infections (Alrajhi et al., 2001), demonstrating the clinical relevance and public health significance of *Aspergillus* research. Furthermore, environmental science emerges as an extra impactful area, with the most cited paper as Alghamdi et al. (2014) published in Science of the Total Environment focusing on environmental monitoring and assessment of *Aspergillus* species. This points to the ecological and environmental health perspectives of research on fungi, at least in the Saudi Arabian context. Food science and agriculture is another major theme, with a number of highly cited papers dealing with applications in food contamination and agriculture. Examples of these papers are Ouf et al. (2015) published in Journal of the Science of Food and Agriculture with 105 citations, and Hashem and Alamri (2010) in Saudi Journal of Biological Sciences with 103 citations, both articles emphasize the economic and food security relevance of studies on *Aspergillus*. It has also branched into biotechnology applications, with recent highly cited papers such as Ali et al. (2020) in Process Biochemistry (76 citations) and Alsaggaf et al. (2020) in Advanced Polymer Technology (60 citations).

A new pattern can be got from the citation data, which show on the impact and recognition of *Aspergillus* research in Saudi Arabia. Whereas the ten top cited papers when evaluated by citations per year contain six were published since 2014, implying that new efforts are still gaining interest. This reflects a growing world curiosity and recognition in the Saudi role in the discipline. Many of the earliest papers from the 1990s still present high normalized citation scores, such as Cameron et al. (1991) at a score of 6.72, clearly highlighting the scientific worth of these works and their influence toward future research pathways even decades later. The normalized citation data also suggest that research impact is increasing overall, as seven out of the ten highest normalized citations correspond to papers published since 2011, implying that recent work is getting cited faster relative to its age. The presence of Hashem and Alamri (2010) from Saudi Journal of Biological Sciences also illustrates the potential for significant impact within national journals, opposing the idea that research is necessarily more visible in international journals for dissemination purposes.

**FIGURE 3:**
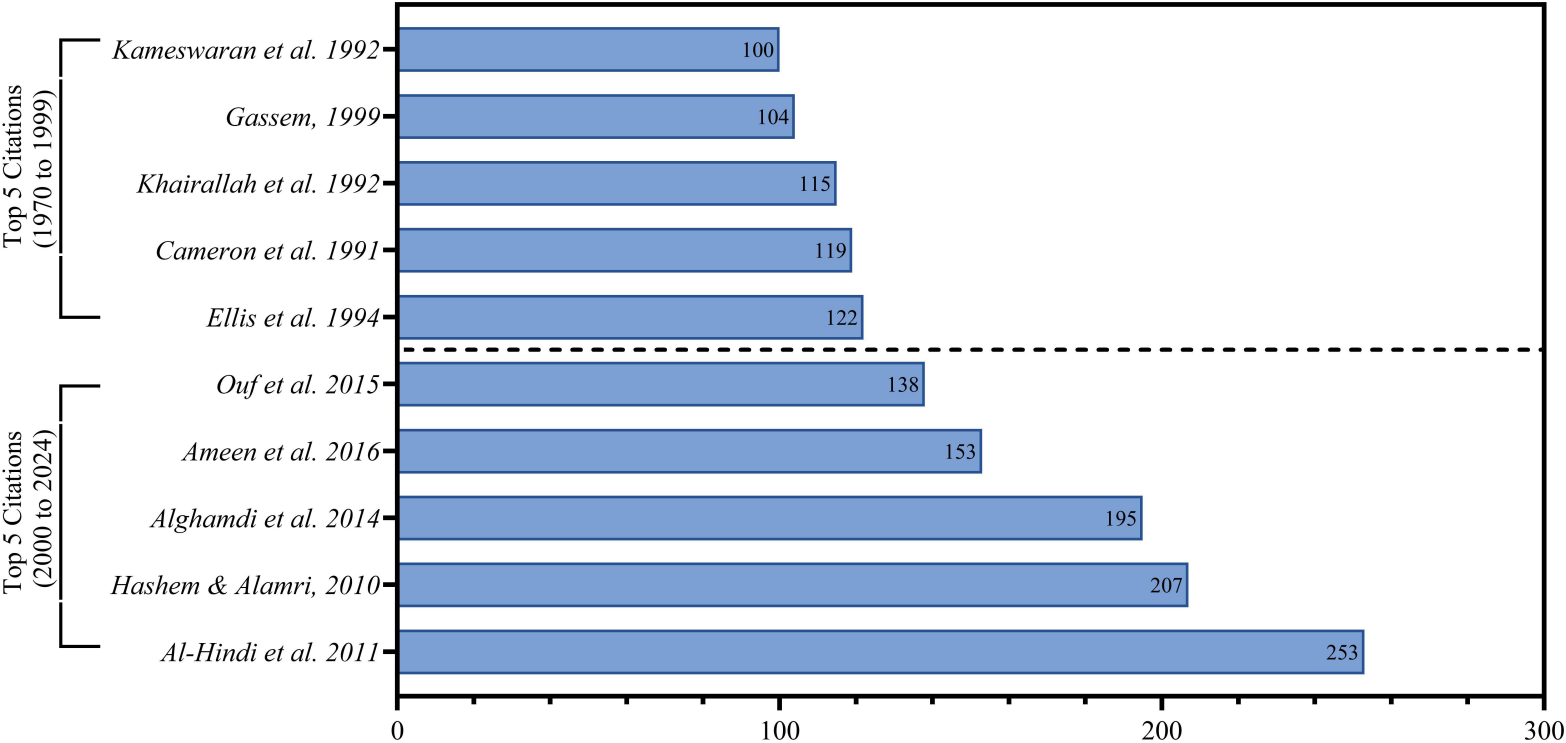
Maximum 5 cited articles according to Google scholar, about scientific production of *Aspergillus* species isolated from Saudi Arabia.

From another different point about citations (Figure 3) shows the Google Scholar citations analysis. Compared to traditional citation databases such as Scopus and WOS, Google Scholar has a broader scope since it captures links to works from a more diverse range of academic and non-academic sources, including conference proceedings, theses, book chapters, and non-indexed journals (Söderlund and Madison, 2015; Gusenbauer, 2019). However, under this alternative scope, there are some major fluctuations on both citations and relative positions of landmark papers. In the entire history of the period 1970–1999, the first five most cited papers according to Google Scholar are Ellis et al. (1994) with 122 cites and Cameron et al. (1991) with 119. By contrast, the most cited current papers (2000–2024) show a strong tendency toward higher citation rates and increased research diversity. The top five in this period are Al-Hindi et al. (2011) with 253 citations, and Hashem and Alamri (2010). A comparison of citation data from Scopus and WOS highlights important methodological considerations. Google Scholar is usually more generous: for instance, Al-Hindi et al. (2011) has 253 citations in Google Scholar and 63 in Scopus/WOS, Alghamdi et al. (2014) have 195 and 138, and Ouf et al. (2015) has 138 and 105. The temporal citation trends also show a large gap in maximum citation attainments between periods, where the highest cited paper for the period 1970–1999 had 122 citations and the highest cited paper for 2000–2024 had 253, being a 107% increase. This indicates a worldwide tendency for an increasing pace of acknowledgment of research and presumably a growing international attention toward *Aspergillus* research in Saudi Arabia. The peak impact period 2010–2016 coincides with a history of emerging interdisciplinary and applied research themes, indicating that impactful research may slightly precede a boom in volume of publications. These trends also highlight the increasing sophistication and global reach of mycology in Saudi Arabia.

**FIGURE 4:**
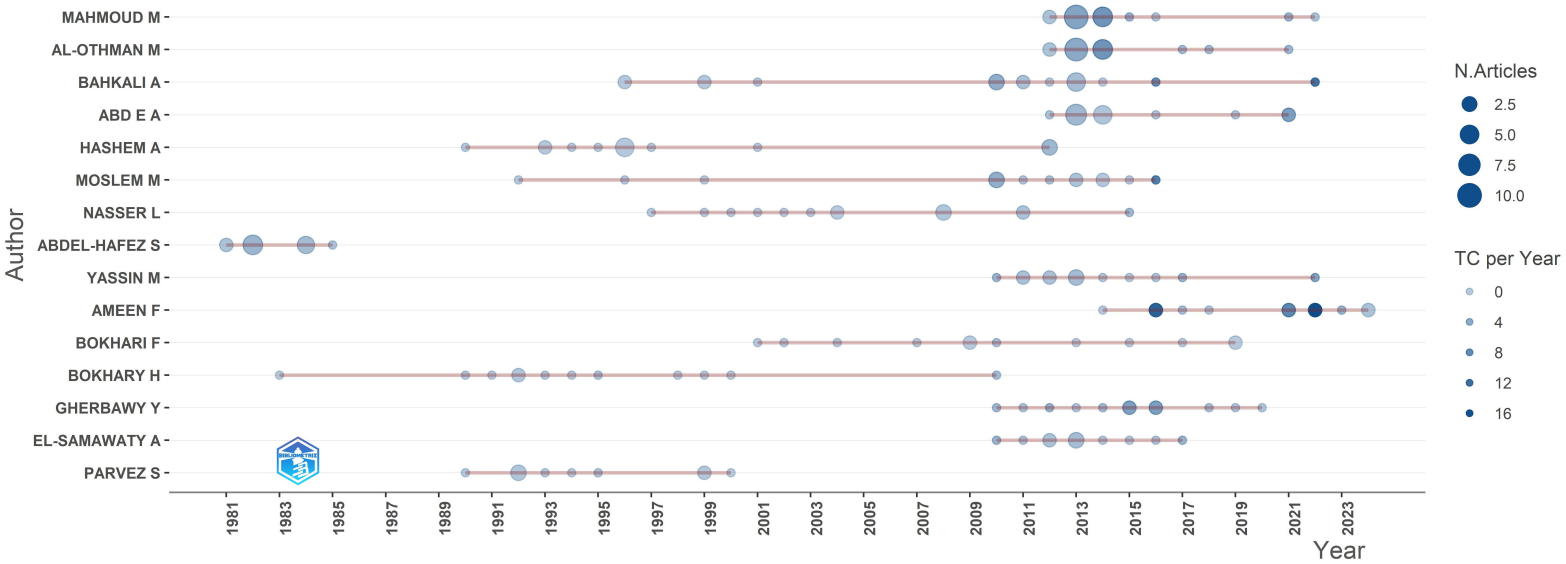
Shows authors production over time in the field of *Aspergillus* species isolated from Saudi Arabia.

The dominance factor is a bibliometric metric that quantifies the significance of researchers within academic subjects. The estimate involves dividing the quantity of multi-authored publications with an author as the lead by the entire number of multi-authored papers (Kumar and Kumar, 2008). This parameter has earned substantial acknowledgment in scholarly literature, as demonstrated by the research of Kinawy et al. (2024). Subsequently, (Figure 4) clearly demonstrates the changing trends of prominent authors in the discipline throughout time. The publishing history indicates a distinct evolution from fragmented, occasional contributions in the 1980s and 1990s to a more vigorous, continuous research environment in the 2000s, especially post-2010. Early pioneers like “Abdel-Hafez S” established foundational work from 1981 to 1985, producing numerous articles that formed the basis for subsequent research. Then, researchers such as “Bokhary H” and “Hashem A” showed ongoing publication endeavors throughout the 1990s, indicating persistent interest during a time when general research output was comparatively limited. A remarkable increase in research productivity transpired post-2010, with the majority of authors exhibiting their peak publication frequency from 2013 to 2018. This timeframe corresponds exactly with the previously recognized peak years of publishing in the comprehensive analysis, affirming that this growth was propelled by heightened engagement from established researchers rather than merely a surge of new contributions. Authors including “Ameen F,” “Bahkali A,” and “Mahmoud M” demonstrate notably high output throughout this timeframe, with numerous papers released in rapid succession. Certain writers, such as “Ameen F,” show significant productivity and notable citation impact, with multiple works attaining considerable citation rates. This indicates research that is both abundant and impactful. Other researchers exhibit varying patterns; some publish fewer articles with consistently high citation rates, while others generate a greater number of publications with middling citation effect. The chronological distribution of significant publications is remarkable. Earlier publications from the 1980s to 1990s often have lower citation rates, however several contemporary articles display exceptionally high citation impact. This implies enhancing the quality and relevance of current research. The concept of “Concentration of Authors’ Contribution” is a recognized metric in bibliometric analysis, as evidenced by Merediz-Solà and Bariviera (2019).

**FIGURE 5:**
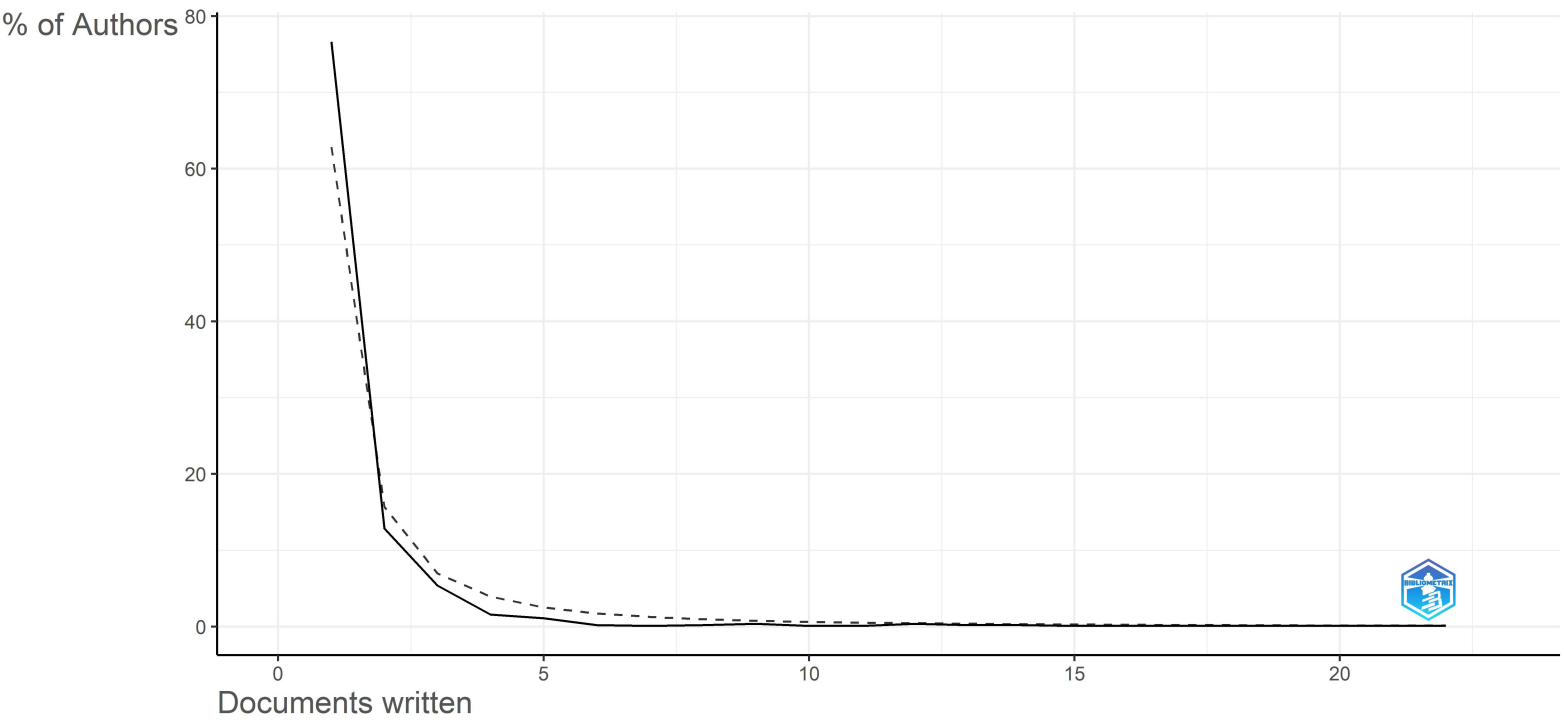
Authors’ productivity research through Lotka’s law.

Figure 5, the graph depicts the utilization of Lotka’s Law (Lotka, 1926) to examine author productivity trends in *Aspergillus* research from Saudi Arabia, demonstrating a typical bibliometric distribution where scientific output is predominantly concentrated among a limited number of researchers. The distribution is markedly irregular, with roughly 65%–80% of authors producing only one publication. The percentage of authors significantly drops to under 20% for individuals with two publications and further diminishes to under 10% for those with three. This persistent decline is evident, since authors generating 10 or more publications constitute a minimal portion of the overall authors demographic. This pattern suggests that most contributors to *Aspergillus* research in Saudi Arabia over the past 50 years are “occasional” authors, generally accountable for a single publication. These individuals likely comprise graduate students, visiting scholars, clinicians documenting unique cases, or researchers whose principal interests are outside the *Aspergillus* domain but who have made singular contributions.

### 3.2 Network analysis

#### 3.2.1 International collaboration networks

**FIGURE 6:**
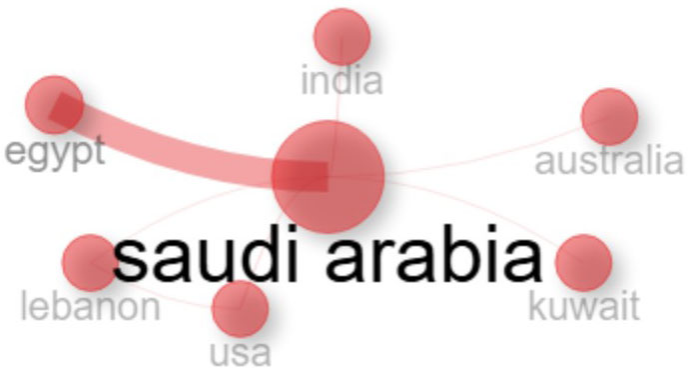
The international collabration network in the field of *Aspergillus* species isolated from Saudi Arabia.

As depicted in (Figure 6) a network visualization showing the international collaboration in *Aspergillus* research involving Saudi Arabia demonstrates a distinct hub-and-spoke model, with Saudi Arabia serving as the central hub. Analysis of collaboration patterns reveals that Egypt is Saudi Arabia’s most significant international research partner in this field. This substantial bilateral collaboration is characterized by extensive co-authorship, joint projects, and institutional linkages, likely influenced by geographic proximity, cultural homogeneity, linguistic similarities, and established academic relationships. Beyond this primary partnership, other notable collaborators include India, Australia, Kuwait, Lebanon, and the USA. While these nations maintain significant collaborative ties with Saudi Arabia, the intensity of these relationships is comparatively moderate when contrasted with the collaboration observed with Egypt. The strong Egypt-Saudi Arabia collaboration is supported by a shared Arabic language, deep historical and religious ties, and major academic and economic initiatives. Further joint institutions like King Salman International University, Egypt’s robust pharmaceutical sector, geographic proximity, and formal bilateral agreements further strengthen this partnership, making it a strategic and sustainable academic alliance. The regional aspect of cooperation within the Middle East and North Africa is further underscored by the fact that Egypt, Kuwait, and Lebanon collectively represent a substantial portion of Saudi Arabia’s top international partners. This regional focus highlights the importance of shared geographical, cultural, and ecological contexts in fostering research pertinent to *Aspergillus* in the region. Furthermore, the collaboration network extends to transcontinental partnerships across North America (USA), Asia (India), and Oceania (Australia). These collaborations with geographically distant partners likely stem from diverse motivations and leverage distinct strengths. The engagement with the USA may facilitate access to advanced research facilities and cutting-edge technologies. Collaboration with India could be driven by shared interests in fungal ecology and agricultural applications, particularly concerning tropical fungi. Partnerships with Australia may capitalize on complementary expertise in arid environment mycology and food safety issues.

**FIGURE 7:**
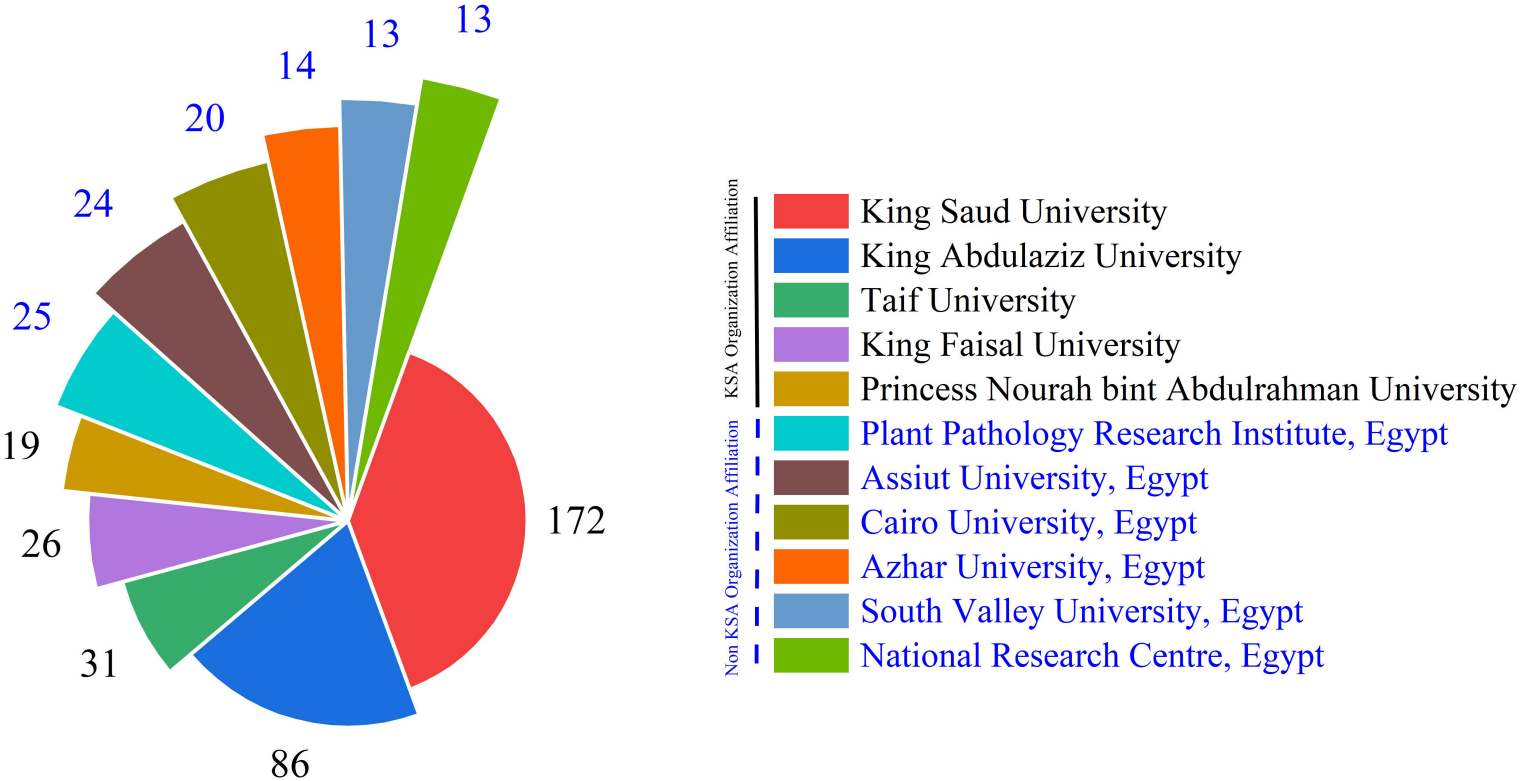
Illustrates the articles number that universities frequently produce regarding the *Aspergillus* species isolated from Saudi Arabia.

Figure 7 also shows the domestic research leadership and international cooperation dynamics that have had an impact on this theme. King Saud University is the main source of research, with 172 papers making up roughly 39% of the total research output that was looked at in this study. This important contribution makes King Saud University the best place in Saudi Arabia for *Aspergillus* research. This may be because of its long history, large research facilities, established mycology departments, and steady funding. As the oldest and biggest school in Saudi Arabia, it is a leader in scientific productivity in the country as a whole. King Abdulaziz University is the second most productive university, with 86 papers, which is around 19% of the total production. Even though it only produces half as much research as King Saud University, this is still a lot of work that makes King Abdulaziz University a major secondary center for mycological studies in Saudi Arabia. These two well-known institutions together account for more than 58% of total research output, showing that there is a lot of research capability in one place. Other Saudi institutions make important but small contributions as Taif University (31 articles, 7%), King Faisal University (26 publications, 6%), and Princess Nourah Bint Abdulrahman University (19 publications, 4%). These five Saudi universities account for more than 75% of all the research done at the institutions. This shows that *Aspergillus* research in Saudi Arabia is mostly done at a small number of well-known academic institutions. In keeping with the fact that Egyptian research institutions have made a big difference in *Aspergillus* research that is important to Saudi Arabia. Six Egyptian institutions are among the most active contributors. These Egyptian institutes together write 109 publications, which is almost 25% of the total amount of research. This large percentage backs up the previous finding that Egypt is Saudi Arabia’s main partner in *Aspergillus* research around the world. The fact that the institutions are spread out over Egypt instead of being in one place shows that there is a broad, multi-institutional collaboration network between the two countries. The spread of Egyptian institutions shows that they make specific contributions. The Plant Pathology Research Institute’s large number of articles (25) shows that they focus on the agricultural and plant disease aspects of *Aspergillus* research. The contributions from Egyptian universities in different parts of the country (Asyut in Upper Egypt, Cairo in the capital region, and South Valley in southern Egypt) show that working with Saudi institutions includes a wide range of areas in Egypt, possibly using different ecological settings and research areas.

#### 3.2.2 The utilization of keywords and co-occurrence network research techniques

**FIGURE 8:**
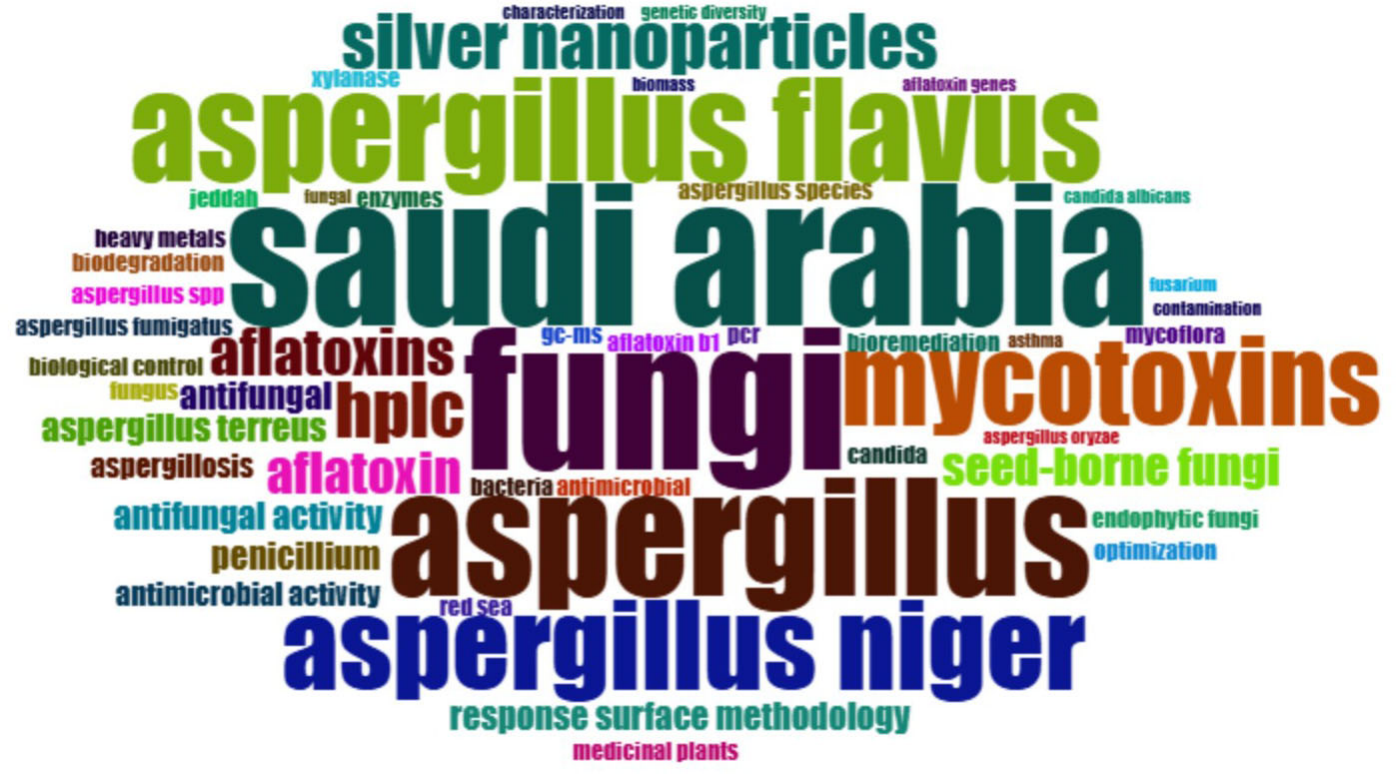
The word cloud visualization provides a comprehensive thematic mapping of *Aspergillus* research in Saudi Arabia.

The KCN, which refers to keyword co-occurrence network, is a common method used in different fields to investigate the structure of knowledge and research trends within literature. It consists of a network where keywords appear as nodes linked to each other based on their co-occurrence in the articles. By examining KCNs, researchers can identify increasing and decreasing areas of research, identify relationships and overlaps within various fields, and improve industrial techniques. The study of KCNs can provide a great deal of understanding of the nature and structure of information in literary texts. It helps academicians, researchers, practitioners, and educators understand where research has lagged and what dominant patterns of knowledge exist in these fields. In addition, keywords are commonly used in scientific studies to represent the main topics of the article because they are brief and specific (Chen et al., 2008). Figure 8 describes a word cloud generated from the author’s keywords. This word cloud functions as a concise and informative tool for summarizing textual information. The dimensions and closeness of each word in the cloud signify its importance, facilitating the rapid recognition of the most prominent and commonly utilized terms (Liao et al., 2019). The phrase “Saudi Arabia” functions as a geographic anchor in the image, highlighting its significance as the research context which we focus on it. The species-specific landscape is predominantly characterized by “*Aspergillus flavus*,” “*Aspergillus niger*,” and the encompassing genus “*Aspergillus*,” with *A. flavus* being the most prominently emphasized, revealing its considerable research focus. This significance is likely related with its function as a significant generator of aflatoxins, hazardous secondary metabolites that pose considerable risks to food safety and human health, especially within the framework of Saudi Arabia’s agricultural systems. Moreover, the obvious inclusion of terms like “fungi,” “mycotoxins,” and “aflatoxins” indicates a significant toxicological focus in the national research program. These expresses constitute a theme cluster indicating ongoing scholarly interest in fungal toxin contamination, particularly in food products prone to aflatoxin buildup. The acronym High-Performance Liquid Chromatography “HPLC” is commonly utilized, indicating its extensive application as a precise analytical method for the detection and quantification of mycotoxins, thereby highlighting the technical complexity of these investigations. Further, “Contamination,” “food,” and “seed-borne fungi” are terms, whose relevance reflects food safety issues, which fit in with existing strategic concerns relevant to Saudi Arabia regarding food security. With a small amount of available land for agriculture, and a trend toward domestic production of foodstuffs, it becomes increasingly important to ensure that both foreign and homegrown products remain free of fungal contamination. In addition to food and toxicological studies, a distinct biotechnology research trajectory is apparent in the word cloud. Words like “silver nanoparticles” and “enzymes” and “biomass” and “optimization” suggest that *Aspergillus* species are investigated in industrial biotechnology. The reference to “silver nanoparticles” would primarily target mycosynthesis, or the fungal production of nanomaterials that may have applications in antimicrobials, catalysis, and biosensing. The terms “bioremediation” and “heavy metals” imply environmental research, on the use of the *Aspergillus* species in the detoxification of polluted environments.

Another critical and crucial theme is the medical and clinical dimensions of *Aspergillus* research are thoroughly represented. The usage of words as “aspergillosis,” “candida,” “antifungal,” and “antimicrobial activity” signifies a strong emphasis on pathogenicity, infection control, and therapeutic advancement. Also, the use of “medicinal plants” suggests a holistic approach, merging ancient herbal treatments with contemporary antifungal research potentially focused on discovering new bioactive molecules to combat fungal infections. Meanwhile the existence of “*Candida albicans*” in conjunction with *Aspergillus* species reveals the need for comparative pathogenic research, presumably focused on discovering shared treatment targets or resistance mechanisms. Additionally the word “asthma” indicates investigations into respiratory health, in particular the aggravating impact of fungal contact on pulmonary conditions, an issue pertinent in both hospital settings and desert, dust-laden areas of Saudi Arabia. Finally, words as “biological control,” “Jeddah,” “Red Sea,” and “endophytic fungi” indicate the ecological and agricultural diversity inherent in *Aspergillus* study throughout Saudi environments. These specify the necessity for research on fungal biodiversity, distribution in terrestrial and marine ecosystems, and the formulation of sustainable agriculture techniques, including the utilization of endophytic or non-pathogenic fungi as biological control agents. Whilst reference to “aflatoxin B1,” a notably strong and carcinogenic form, highpoints the intricacy and thoroughness of toxicological research, particularly with basic food commodities like nuts and grains that are significantly pertinent to Saudi food systems.

**FIGURE 9:**
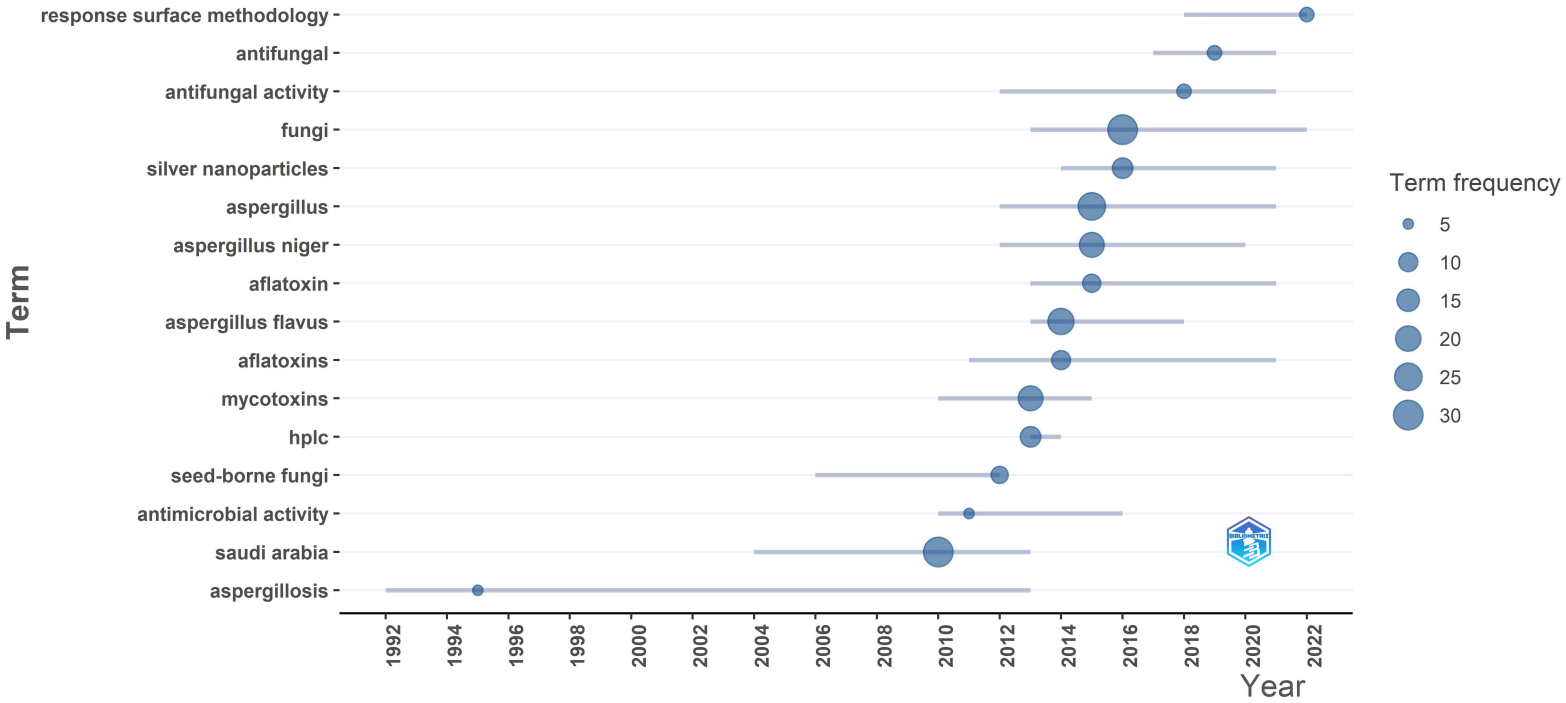
*Aspergillus* spp. isolation from Saudi Arabia research trending topics over time.

Main trends are illustrated in (Figure 9), the trend themes visualization shows how the themes in *Aspergillus* research in Saudi Arabia have changed over time, from 1992 to 2022. This historical study shows how research goals have changed over the past 30 years, how new areas of focus have been added, and how the importance of certain themes has changed. It gives us a look at how the discipline has evolved and adapted to meet the needs of science and society. “Aspergillosis” is one of the first major topics that stands out, with activity continuing from 1992 to 2013. This shows that the main clinical focus of Saudi research is on understanding and treating *Aspergillus*-related diseases, especially in people with weak immune systems. But after 2013, “aspergillosis” becomes less important compared to other study goals. The years 2012–2018 were a very productive time for research and subject development. During this time, words like “*Aspergillus*,” “fungi,” “*Aspergillus flavus*,” “*Aspergillus niger*,” “mycotoxins,” and “aflatoxins” are at their most active. This change in themes goes along with a general rise in the number of publications, which shows that research infrastructure is getting better and that scientists are purposefully looking into areas outside of clinical mycology. Moreover from 2012 to 2018, *Aspergillus flavus* was the subject of a lot of research since it is the main source of aflatoxins and is therefore very important for food safety. On the other hand, *Aspergillus niger* has been studied for a longer period of time, with the most study happening between 2014 and 2018. The fact that both species have bubbles of the same size shows that they are both of equal academic interest. This shows how important they are as members of the *Aspergillus* genus that are useful in both medicine and agriculture. The word “*Aspergillus*” has the highest bubble size of all the species-related phrases. This means that there is a lot of study at the genus level that goes beyond species-specific limitations.

#### 3.2.3 Thematic map

**FIGURE 10:**
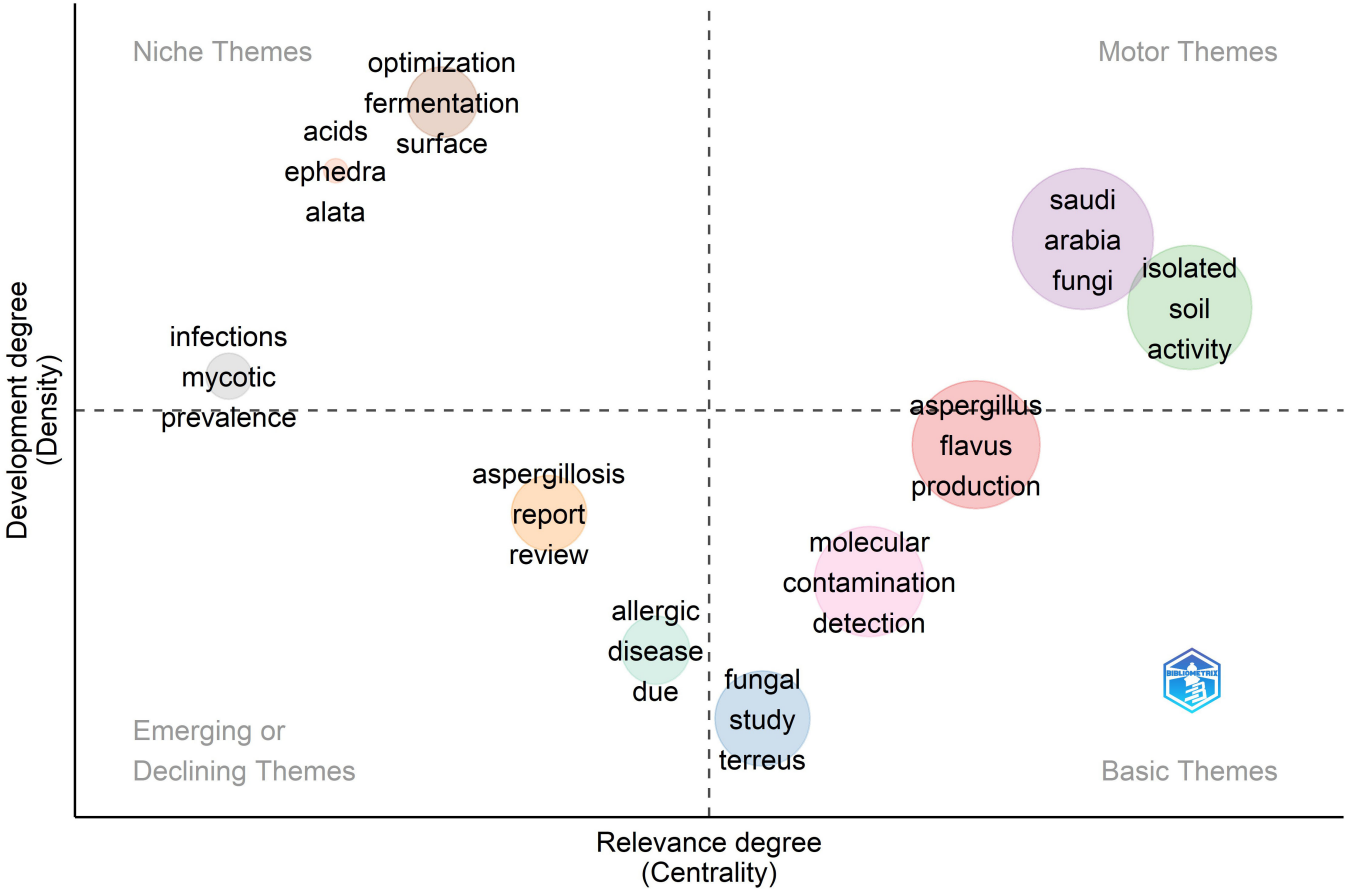
*Aspergillus* spp. isolation from Saudi Arabia research thematic/strategic map.

The strategic thematic map (Figure 10) offers a comprehensive analytical depiction of the Saudi Arabian *Aspergillus* research landscape, organizing thematic clusters according to two essential dimensions, the level of development (density) on the vertical axis and thematic significance (centrality) on the horizontal axis. This two-dimensional framework enables the assessment of thematic maturity and strategic positioning across many research domains within the discipline. The motor topics, distinguished by extensive development and fundamental relevance to the research landscape, are positioned in the upper-right quadrant. These themes constitute the conceptual and methodological bedrock of Saudi *Aspergillus* research, exerting a considerable influence on the overall structure and progression of the field. The primary driving topic cluster integrates the terms “Saudi Arabia,” “fungi,” and “isolated,” collectively depicted by a prominent purple circle. The reference to “soil activity” signifies foundational research on *Aspergillus* species with respect to bioremediation, soil fertility enhancement, and biocontrol. Although these applications are not frequently overtly identified, they exemplify the cluster’s practical significance in environmental and agricultural sectors. This cluster functions as a principal axis, anchoring the research both geographically and biologically. The heightened centrality and density indicate that the Saudi context and broader fungal isolation efforts have been thoroughly explored, thereby establishing a robust thematic foundation for the broader research domain. A second principal thematic cluster encompassing “soil” and “activity” underscores the ecological aspect of *Aspergillus* study. These words indicate an emphasis on soil as a principal habitat and on biological or enzymatic activity as a key research subject. This cluster, albeit marginally less developed than the Saudi Arabia/fungi cluster, maintains a pivotal position that highlights its continued significance and potential for additional growth. The core themes, characterized by great importance but comparatively modest development, are situated in the lower-right quadrant. These fundamental research subjects, although vital to the field, seem to require further methodological enhancement or conceptual clarification. A notable instance in this quadrant is the “*Aspergillus flavus*” and “production” cluster (in red), highlighting the species’ significance due to its aflatoxin biosynthesis and its essential role in food safety studies. The positioning indicates that although *A. flavus* is a fundamental topic, there are still chances to enhance scientific comprehension and refine research approaches, especially in the Saudi setting. Additional fundamental topics encompass “molecular,” “contamination,” “detection,” and “fungal study.” These phrases signify fundamental components of the research agenda, encompassing genetic and molecular investigations, food and environmental pollution, and fungal characterization. The designation of “molecular” as a fundamental rather than operational theme underscores a relative underutilization of molecular tools and procedures, indicating untapped potential for furthering molecular mycology in the region. The placement of “contamination” and “detection” highlights the ongoing emphasis on food safety and mycotoxin surveillance areas that are still pertinent yet might gain from methodological advancements and the incorporation of emerging technology. The upper-left quadrant contains niche themes characterized by significant development (density) but low importance (centrality). These specialist subjects have undergone rigorous study endeavors but are less incorporated into the wider research framework. Terminology such as “optimization,” “fermentation,” “acids,” “Ephedra,” and “Alata” resides within this quadrant, indicating study trajectories that are both technically sophisticated and culturally distinct. The terms “optimization” and “fermentation” indicate focused biotechnological research, such as enzyme synthesis or metabolite augmentation, whereas “Ephedra” and “Alata” presumably relate to medicinal plants investigated for antifungal properties or bioactive chemical generation. These specialized areas illustrate unique Saudi scientific contributions, perhaps influenced by local biodiversity, regional health issues, or strategic government priorities in biotechnology and environmental sustainability. Despite being well-developed in their specific areas, their low centrality indicates a necessity for enhanced conceptual or methodological integration within the wider *Aspergillus* research framework. Closing this gap could amplify the significance and interdisciplinary applicability of these specialized research initiatives.

#### 3.2.4 Ecological distribution of *Aspergillus* isolates

**FIGURE 11:**
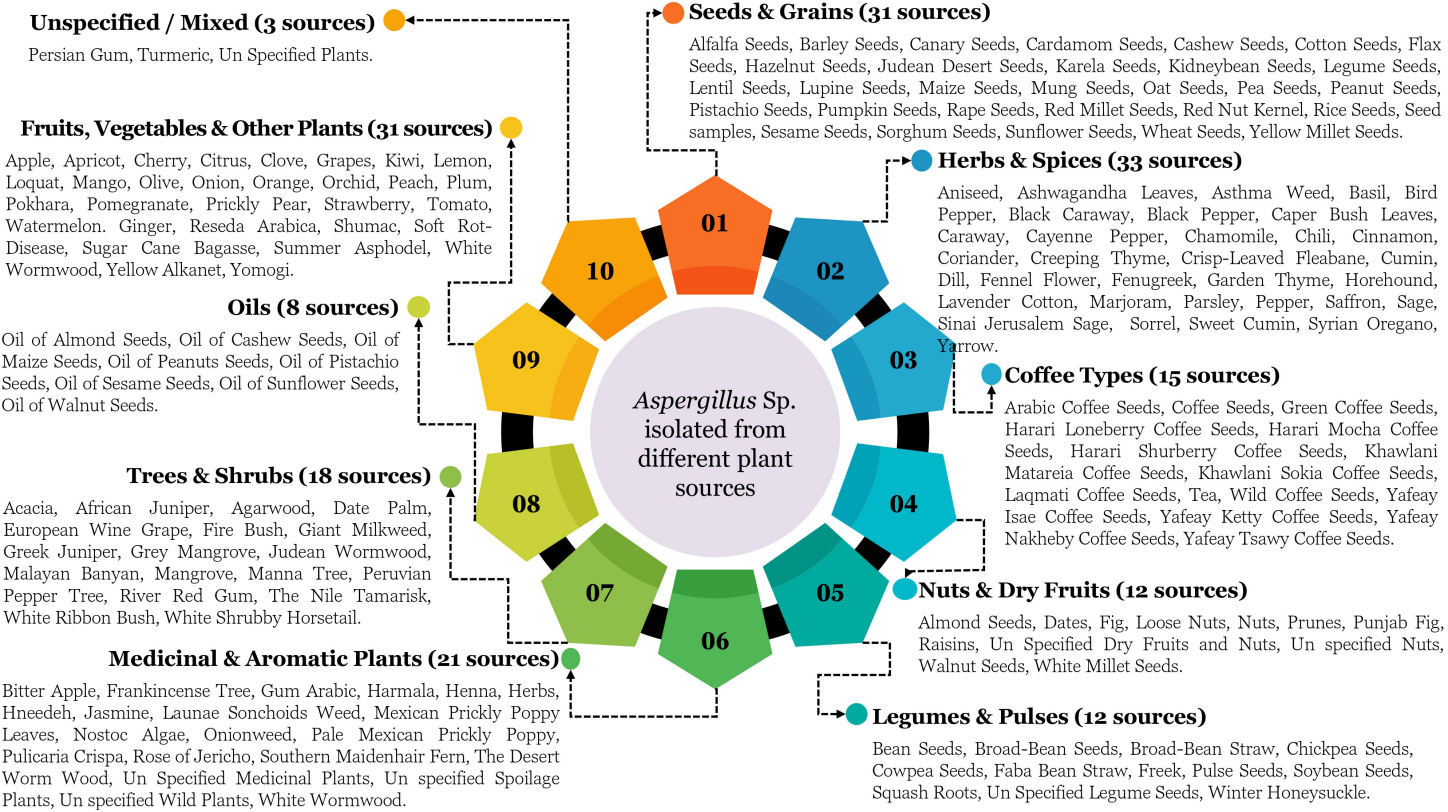
Shows a detailed mapping of *Aspergillus* isolation from plant sources in Saudi Arabia.

Upon examining the study publications, we discovered that all identified *Aspergillus* spp. may be classified into ten primary sources. Figure 11 delineates a thorough classification of plant-based sources from which *Aspergillus* species have been isolated in Saudi Arabia, representing the primary sources with 184 instances of *Aspergillus* identified throughout 351 research. Seeds and grains serve as a main isolation source, with 31 recognized kinds, including economically significant crops (wheat, rice, barley), oil-producing seeds (sunflower, flax), and locally relevant types (Jirdaan Desert Seeds, Kulneybean Seeds). The extensive presence of various seed types underscores the critical role of *Aspergillus* species as seed-borne fungus, with considerable consequences for food safety (possible mycotoxin contamination) and agricultural production (seed viability and germination). The extensive distribution throughout cereals, pulses, and oilseeds signifies non-selective colonization abilities across diverse seed compositions and architectures. Fruits, vegetables, and other plants represent a significant isolation category, encompassing 31 separate sources, including common fruits (apple, citrus, grapes), regionally significant food (dates, pomegranate), and specialty plant materials (*Reseda arabica*, wormwood). This broad distribution illustrates the capacity of *Aspergillus* species to inhabit plant materials with diverse sugar concentrations, acidity levels, and protective chemicals. The presence in both fresh market produce and specialist plant materials indicates adaptability to cultivated and wild plant sources in Saudi Arabian environments.

Herbs and spices represent a notably varied category for isolation, with 33 verified sources, marginally exceeding seeds/grains and fruits/vegetables. This category includes commonly used culinary herbs (basil, cumin, sage), medicinal plants (ashwagandha, fenugreek), and regionally significant spices (caraway, black caraway). The prominent representation of herbs and spices signifies their vulnerability to *Aspergillus* colonization, which may influence food quality and the efficacy of medicinal plants. The variation in essential oil compositions among herbs suggests adaptation to distinct plant biochemical defenses. Coffee varieties constitute a separate classification with 15 unique sources, highlighting the economic and cultural importance of coffee within the Saudi Arabian context. The differentiation among specific coffee types (Harari Lonberry, Khawlani, Yaffay Kerty) exemplifies a comprehensive assessment of *Aspergillus* contamination in this highly regarded product. The association of tea with coffee variants signifies an extensive examination of caffeinated beverage sources within the research scope. Nuts and dried fruits (12 sources) and legumes and pulses (12 sources) form moderately sized yet economically significant isolation categories. The documented nut varieties, such as almonds, pistachios, and walnuts, are particularly noteworthy from a food safety perspective due to their susceptibility to aflatoxin contamination. Legumes such as chickpeas and broad beans are essential protein sources in the regional diet, rendering their *Aspergillus* colonization patterns relevant to food security and safety concerns. Medicinal and aromatic plants constitute a prominent isolation group, comprising 21 identified sources, including traditional medicinal herbs (harmala, henna), fragrant plants (jasmine, frankincense tree), and culturally significant plants (gum arabic, white wormwood). This category underscores the intersection of traditional medicinal practices and potential issues related to fungal infection. The extensive involvement of several medicinal plants indicates both a research interest in protecting these unique resources and concerns regarding the impact of *Aspergillus* metabolites on their medicinal properties. Trees and shrubs form a notable category with 18 isolation sources, encompassing economically significant species (date palm, tufted pine), ecologically important plants (gray mangrove, acacia), and culturally significant species (frankincense tree). This category demonstrates the capacity of *Aspergillus* species to inhabit woody plant materials characterized by diverse resin concentrations, structural attributes, and environmental adaptations. The presence of both indigenous desert-adapted species and non-native plants illustrates the fungal adaptability to various plant hosts within the Saudi ecosystem. Oils derived from botanical sources constitute a distinct category with eight recognized origins, all obtained from commercially significant seeds (almond, cashew, pistachio, sesame, sunflower, walnut). This category presumably represents processed agricultural products as opposed to raw plant materials, indicating a necessity for research on *Aspergillus* contamination throughout the agricultural supply chain, from raw inputs to finished goods.

**FIGURE 12:**
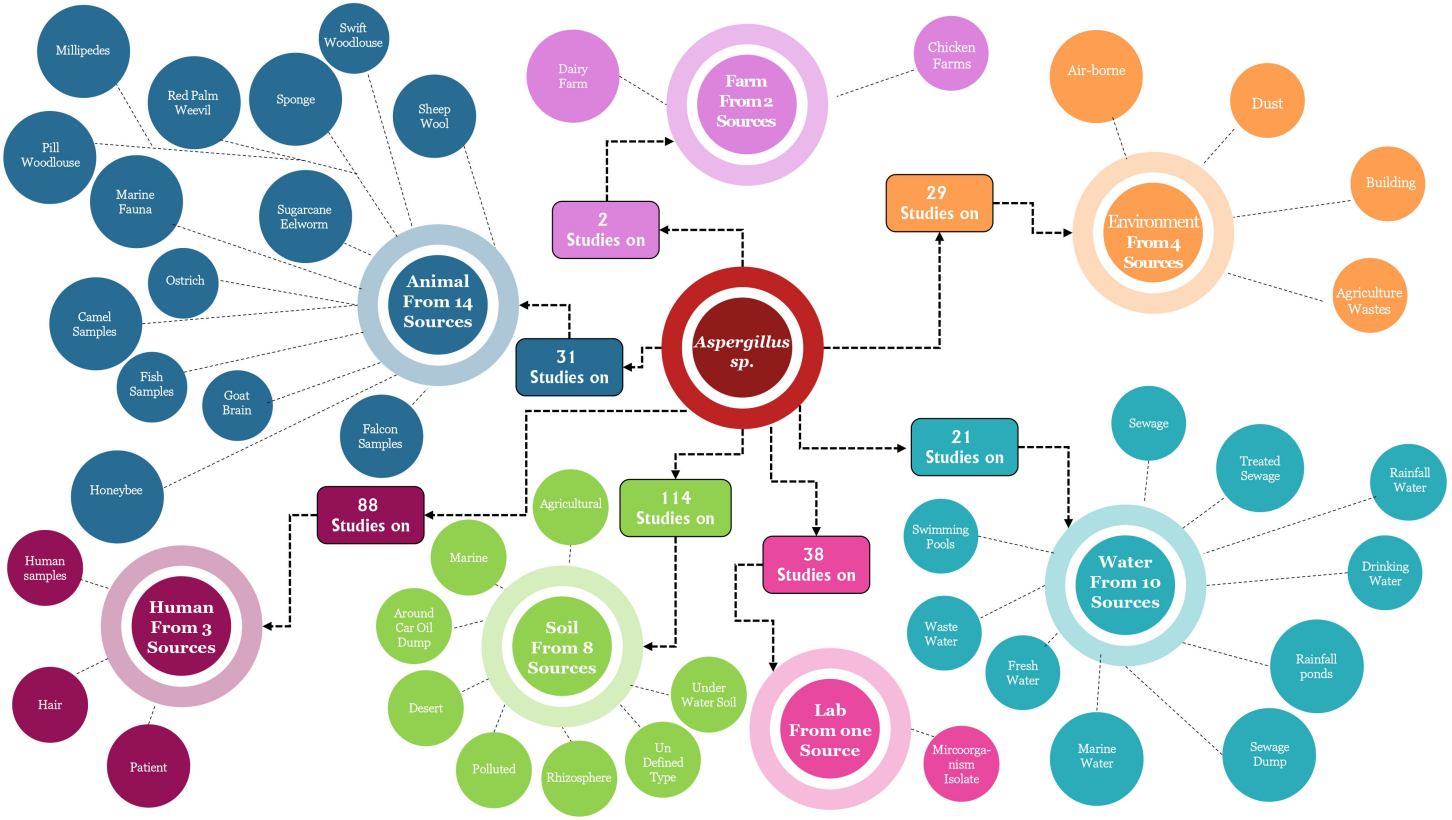
Depicts a detailed mapping of *Aspergillus* isolation sources in Saudi Arabia.

In addition, Figure 12 presents another detailed mapping of *Aspergillus* isolation sources in Saudi Arabia. Soil is the primary isolation source, with 114 studies constituting around 36% of the overall research effort. This significant emphasis on soil habitats underscores the essential ecological function of *Aspergillus* species as soil-dwelling organisms within Saudi Arabian terrestrial ecosystems. The soil group is further subdivided into specialized niches such as desert conditions, contaminated soils, rhizosphere zones, subaqueous soil, and oil-refined typic habitats. This distinction illustrates the exceptional resilience of *Aspergillus* species to numerous soil conditions, including the harsh settings typical of Saudi Arabia’s varied landscapes. Animal sources are the second most prominent isolation group, with 51 investigations into over 14 different animal-associated substrates. The variety of animal sources is notably impressive, encompassing domesticated animals (camel samples, ostrich), marine organisms (fish samples, marine fauna), insects (millipedes, pill woodlouse), and agricultural products (sheep wool, sugarcane). The extensive presence of *Aspergillus* species in both terrestrial and aquatic animals underscores their significant function as commensals and potential pathogens in Saudi Arabian animal populations. Human sources comprise 88 papers, indicating a significant research commitment to the medical and public health aspects of *Aspergillus* ecology. The identified human sources, including patient samples, hair, and human brain, underscore the clinical focus of this research, emphasizing harmful interactions above commensal associations. The extensive array of research, notwithstanding the scarcity of particular sources, indicates a rigorous medical examination of human aspergillosis cases, presumably concentrating on clinical symptoms, treatment outcomes, and epidemiological trends. Water sources represent a crucial biological niche, evidenced by 31 studies conducted across 10 different aquatic settings. The distribution encompasses drinking water, rainwater ponds, sea water, sewage, treated sewage, recycled water, wastewater, freshwater, sewage disposal, and swimming pools. This extensive examination of aquatic habitats illustrates the adaptability of *Aspergillus* species to various water conditions, ranging from drinkable supplies to severely polluted areas. The presence in drinking water sources holds specific public health implications, indicating possible exposure pathways beyond the more widely acknowledged airborne spore transmission. Environmental sources comprise 23 investigations spanning four diverse media: airborne, dust, building, and agricultural waste. The very little quantity of environmental studies in relation to soil investigations indicates a research opportunity, especially considering the established significance of airborne transmission for *Aspergillus* spores. The incorporation of built environment sources (such as buildings and agricultural waste management facilities) acknowledges the significance of human-altered settings as critical homes for *Aspergillus* in the context of Saudi Arabia. Farm sources are a specific domain of research, with 7 studies that analyze 2 separate agricultural environments: dairy farms and poultry farms. This focused research presumably addresses food safety and agricultural production issues associated with *Aspergillus* contamination in livestock operations. The emphasis on dairy and poultry, as opposed to other livestock sectors, may suggest either a heightened susceptibility of these activities to *Aspergillus* infection or a greater economic significance within the Saudi agricultural framework. Remaining other sources as food sources contained 32 sources for *Aspergillus* spp. induced in 39 investigations, and industrial sources comprised 15 sources for 18 investigations, as detailed in supporting data (Table 1).

### 3.3 Analysis of taxonomic richness and diversity

**FIGURE 13:**
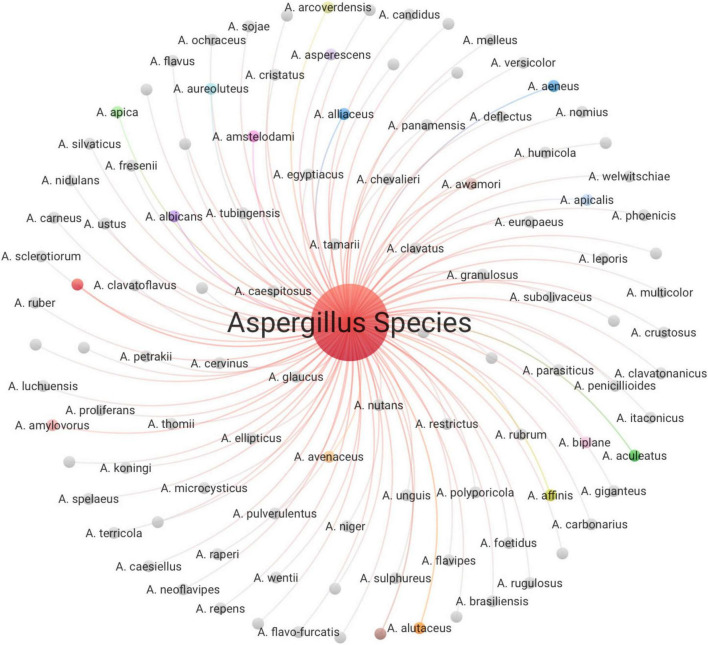
Illustrated all detected *Aspergillus* isolates in Saudi Arabia.

We identified 108 distinct isolates of *Aspergillus* spp. from Saudi Arabia, as illustrated in Figure 13. This notable taxonomic variety accounts for around 23.8% of the globally recognized *Aspergillus* species, suggesting that Saudi Arabian ecosystems contain a significant share of the global diversity within this crucial fungal genus. It identifies several species of economic and medical significance, *A. flavus* notable entity, underscoring its importance as a principal aflatoxin generator and prevalent contamination of agricultural commodities. *A. niger* a significant entity, recognized as one of the most frequently isolated *Aspergillus* species globally. *A. parasiticus* a significant aflatoxin generator that is identified as a unique entity, crucial in food safety research, especially concerning contamination of nuts and seeds. *A. fumigatus* a clinically important species that is the principal etiological agent of invasive aspergillosis in immunocompromised individuals. Its existence among the detected species relates to the medical research aspects highlighted in prior assessments. Furthermore, several rare and potentially unique species of *Aspergillus* have been identified, potentially indicating specific adaptations to the environment of Saudi Arabia or efforts in targeted isolation: *A. sclerotiorum*, *A. spelaeus*, *A. welwitschiae*, and *A. itaconicus*. The presence of these relatively rare species underscores the need for expansive sampling and identification procedures that extend beyond the commonly observed *Aspergillus* species. Their detection may suggest the existence of particular biological niches within Saudi habitats or the use of sophisticated identification tools that aid in the identification of cryptic species. *A. spelaeus*, a species adapted to cave environments and tolerance of nutrient-deficient, humid conditions, has been isolated from various cave systems globally. Its occurrence in Saudi datasets implies the existence of stable, specialized subterranean habitats conducive to such taxa. Also, *A. itaconicus*, initially isolated from salted prune juice, demonstrates adaptation to high-salt, high-temperature, and acidic environments, which correspond to the extreme ecological niches present in some regions of Saudi Arabia. Their identification in Saudi research emphasizes the ecological diversity of the country and the potential for discovering lesser-studied *Aspergillus* species that have adapted to unique or extreme environments.

**FIGURE 14:**
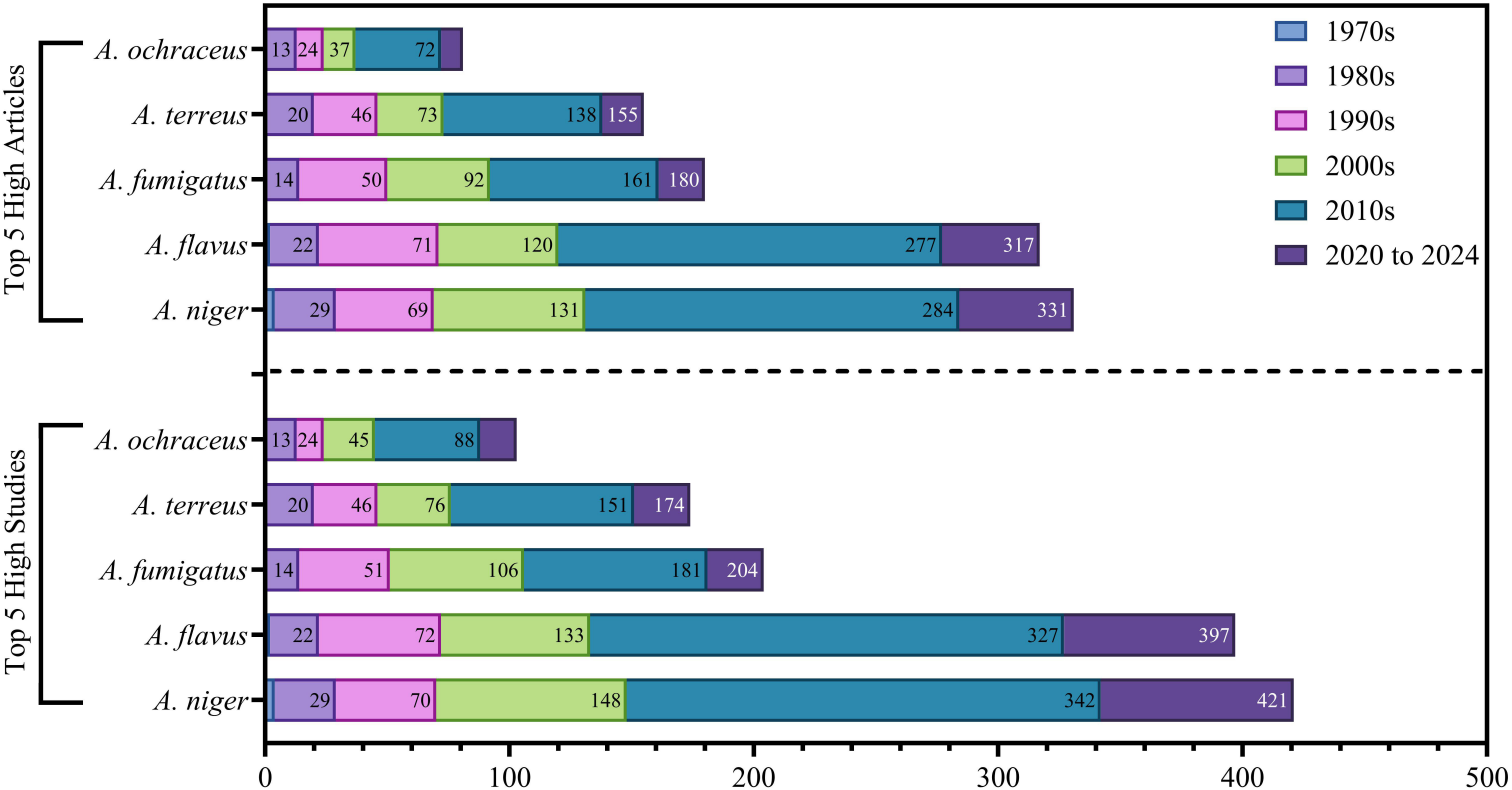
Illustrates the five principal strains that were isolated and utilized in various experiments and publications.

The frequently isolated species, *A. niger*, *A. flavus*, *A. terreus*, *A. fumigatus*, and *A. ochraceus*, were regularly recorded in several studies, as determined in (Supplementary Figures 1–5). These species were obtained from diverse environmental and biological sources, underscoring their ecological adaptability and prominence within the Saudi Arabian context. Figure 14 shows the progressive sequence of *Aspergillus* research in Saudi Arabia over five decades, highlighting distinct growth patterns and strategic emphasis on key species all other species are detected in (Supplementary Table 1). *Aspergillus niger* leads in research production, with 421 studies and 331 published articles, indicative of its extensive agricultural and industrial significance. *Aspergillus flavus* is ranked second (297 studies, 317 papers), highlighting its significance in food safety research as a principal aflatoxin generator. *Aspergillus fumigatus* is supported by 204 research and 180 articles, highlighting its clinical importance as a predominant human pathogen. *Aspergillus terreus* (174 studies, 155 publications) demonstrates growth patterns analogous to *A. fumigatus* but at diminished levels, whereas *Aspergillus ochraceus* (105 studies, 81 articles) shows the least productivity, roughly 25% of the predominant species. Temporal study identifies six unique developmental periods. The 1970s was a groundbreaking period, characterized by merely seven studies, reflecting the embryonic state of mycological research infrastructure. Foundational expansion transpired in the 1980s (93 studies), marked by methodological uniformity and taxonomic classification. Enhanced institutional capability developed in the 1990s (170 studies), coinciding with improved infrastructure and acknowledgment of *Aspergillus’s* multifaceted effects. The 2000s were a period of maturation (260 studies), characterized by methodological variety and collaborative networks. The 2010s experienced significant increase (551 studies, +112% over the previous decade), along with national research plans that improved financing, infrastructure, and international collaborations. This phase facilitated simultaneous progress in agricultural, clinical, environmental, and biotechnological applications, illustrating a comprehensive research ecosystem. The sustained production from 2020 to 2024, comprising 212 studies over 4.5 years, illustrates good stability at heightened output levels, signifying institutionalized knowledge and durable research methodologies. The steady growth among all species throughout the 2010s, coupled with sustained productivity after 2020, highlights Saudi Arabia’s shift from rapid expansion to a developed, multidisciplinary research environment. This trajectory emphasizes the intentional incorporation of *Aspergillus* research into national priorities, guaranteeing sustained capability to tackle new concerns in public health, food security, and biotechnology.

## 4 Discussion

### 4.1 Chronological progression of *Aspergillus* investigation

On second thoughts chronologically we can divide the development of the current research point of *Aspergillus* in Saudi Arabia into four main stages. The first studies by Yaman and Blan (1971) and Abdel-Hafez (1981) 1982 provided foundational insights through documenting the prevalence of *Aspergillus* in both agricultural land and arid soil environments. These seminal investigations identified *Aspergillus* as a significant constituent of the native mycoflora in Saudi Arabia. Throughout the developmental phase of the 1970s and 1980s, studies extensively examined *Aspergillus* within desert soil contexts, along with thermophilic fungi, thermotolerant fungi, osmophilic fungi, cereal grains, and airborne fungal spores in the region of Taif. This exploratory research was instrumental in establishing foundational data concerning the distribution of *Aspergillus* species within the Saudi Arabian ecological setting. Food safety and agricultural impact concerns exploded into research especially in the 1990s and 2000s. This is exemplified by studies conducted by Al-Julaifi et al. (1996) for patulin in barley; Abdel-Gawad and Zohri (1993) for mycotoxins in nuts; and Bokhari (2007a) for mycotoxins in coffee beans. This evolution can be seen from studies like Gherbawy et al. (2020) dealing with molecular characterization of the ochratoxigenic fungus; Ameen et al. (2018) dealing with fungi detected in bottled water; and Sajer et al. (2024) on the antibacterial and anticancer effects of *Aspergillus* from Sabkha marsh.

### 4.2 Ecological distribution and environmental adaptability

A central theme in these investigations is the extraordinary ecological adaptability of *Aspergillus* species in Saudi Arabia. The research demonstrates that *Aspergillus* flourishes in a wide array of settings. A multitude of studies record the presence of *Aspergillus* across diverse soil types (Abdel-Hafez, 1982), encompassing desert soils, agricultural soils (Abdulla and El-Gindy, 1987), oil-contaminated soils (Bokhary and Parvez, 1993), indoor environments (Bokhary and Parvez, 1995), and Sabkha (salt marsh) soils (Sajer et al., 2024). In opposition to conventional perceptions of *Aspergillus* as predominantly terrestrial fungi, numerous studies have recorded their occurrence in aquatic habitats, including freshwater (Arif, 1999), bottled water (Ameen et al., 2018), swimming pools (Najjar et al., 2019), drinking water systems (Gashgari et al., 2013), and groundwater. The findings have substantial public health implications, indicating that water sources may act as vectors for *Aspergillus* exposure. Research conducted by Saati and Faidah (2013) recorded *Aspergillus* contamination in multiple drinking water sources in Makkah, underscoring potential hazards, particularly for immunocompromised patients. A multitude of studies have concentrated on *Aspergillus* in relation to various plants, encompassing the phyllosphere and phylloplane of wheat (Abdel-Hafez, 1981), dates (Abu-Zinada and Ali, 1982; Al-Sheikh, 2009), cereal grains (Abdel-Hafez, 1984; Yassin et al., 2010), spices (Hashem and Alamri, 2010), and coffee beans (Bokhari, 2007b). These interactions vary from superficial contamination to endophytic partnerships. Recent research, such as that conducted by Gherbawy and Gashgari (2014), has examined endophytic *Aspergillus* from *Calotropis procera*, focusing on potential advantageous features, including antibacterial activity. The study further records *Aspergillus* in relation to both animals and humans, encompassing animal hair (Bagy and Abdel-Mallek, 1991), sheep wool (Abdel-Gawad, 1997), camel infections (Al Hizab, 2014), human clinical specimens (Naim et al., 1989), and hospital settings (Tabbara and Al Jabarti, 1998). The variety of different habitats illustrates the remarkable adaptability of *Aspergillus* species and their capacity to inhabit nearly any ecological niche present in Saudi Arabia. The accelerated aridification of Saudi Arabia, manifested by rising temperatures and diminishing precipitation, is transforming the biological dynamics and distribution of *Aspergillus* species. Many *Aspergillus* species demonstrate considerable thermotolerance (e.g., *A. fumigatus* thrives at temperatures exceeding 50°C) and xerotolerance (e.g., *A. penicilloides* thrives with minimal water activity), equipping them to endure and even thrive in arid environments (Korfanty et al., 2023; Pócsi et al., 2024). Nevertheless, the climatic impacts are species-specific: while some, such as *A. terreus* and *A. flavus*, may expand their distribution, others may not fare as well. Desert-adapted species found in Saudi Arabia, including *A. niger*, *A. flavus*, and *A. ochraceus*, possess physiological traits (e.g., heat-shock proteins, solute accumulation) that enable survival under extreme conditions (Alotaibi et al., 2020; Sajer et al., 2024). Aridification might modify soil microbial dynamics, potentially favoring fungi over bacteria, while the geographical heterogeneity across Saudi landscapes will lead to varied effects (Riedling et al., 2024). These trends underscore the necessity for targeted monitoring of key species and the advancement of research into thermotolerance and environmental adaptability, to guide efforts in biodiversity conservation and biotechnological application in the context of ongoing climate change.

### 4.3 Presence of main species and research prioritization

Upon examining the 520 articles, it was determined that Saudi Arabia has documented 108 *Aspergillus* species; however, the actual diversity may be overestimated or underestimated, this is particularly true in older literature, due to potential misidentification of morphologically similar species, especially in studies conducted between 1970 and 1990, representing approximately 23.8% of the total 453 *Aspergillus* species (Visagie et al., 2024). This diversification illustrates the resilience of *Aspergillus* and the evolution of research procedures, which have progressed from fundamental morphological identification to contemporary molecular and polyphasic techniques. Notwithstanding this diversity, research has continuously concentrated on five principal species, demonstrating strategic prioritizing in accordance with national agricultural, industrial, and health objectives. *Aspergillus niger* is predominant with 421 studies and 331 articles, experiencing a 48-fold increase from the 1971s to the 2010s. Its significance arises from its dual function in biotechnology (e.g., enzyme synthesis) and as a contaminant impacting food preservation. *Aspergillus flavus* is associated with 297 studies and 317 articles, experiencing an 82-fold increase over five decades. Their dynamic significance stems from the creation of aflatoxins, extremely deadly carcinogens rendering them a primary issue for food safety and public health, particularly in a nation heavily dependent on food imports and the development of indigenous agriculture. *Aspergillus fumigatus*, utilized in 204 research and 180 articles, is prioritized due to its clinical significance, particularly as a predominant cause of invasive aspergillosis in immunocompromised individuals. The research trajectory corresponds with the advancement of sophisticated medical treatment in Saudi Arabia. *Aspergillus terreus* (174 studies) and *A. ochraceus* (105 studies) complete the top five, each holding specific importance in clinical and food safety domains, respectively.

### 4.4 Different sources for the isolated strains

Over the past five decades, *Aspergillus* scientific research in Saudi Arabia has acquired a significant transformation, reflecting the extensive evolution of the country’s scientific and institutional view. The 1971s and 1980s consider the influential stage, with minimal output (typically 0–2 publications annually) and a narrow clinical focus primarily on *A. niger* and *A. flavus*. Research efforts during this time were limited to a few pioneering scientists working with rudimentary resources. The 1990s through the early 2000s marked a period of steady development, with annual output increasing to 3–13 publications. This phase consider the development of agricultural and environmental research alongside continued clinical work, and institutions such as King Saud University began expanding their research capacities. From 2006 to 2012, *Aspergillus* research played a growth phase characterized by methodological advances, including standardized mycotoxin detection and initial use of molecular techniques. During this time, more universities became actively involved, contributing to a noticeable increase in publication output (13–27 articles annually). The most dramatic expansion occurred between 2013 and 2018, with output peaking at 41 publications in a single year an 800-fold increase from the earliest years. This rise matched national investment in research, higher education expansion, and increased international collaboration (Shehatta and Mahmood, 2016). Multidisciplinary integration became a hallmark of this phase, combining clinical studies on *A. fumigatus*, agricultural research on *A. flavus* and mycotoxins, environmental assessments of soil and water, and biotechnological applications involving *A. niger*. Institutionally, the research landscape evolved from being highly centralized to a more distributed yet focused network. King Saud University emerged as the national leader with 172 publications (39%), followed by King Abdulaziz University with 86 publications (19%). Taif University, King Faisal University, and Princess Nourah bint Abdulrahman University collectively contributed another 17%, creating a strong core of five institutions accounting for about 75% of all national output. International collaborations further strengthened Saudi Arabia’s research ecosystem, particularly with Egyptian institutions (109 publications, ∼25%), and additional partnerships with India, Australia, Kuwait, Lebanon, and the USA facilitated cross-border knowledge exchange and methodological enrichment. This progressive institutional and disciplinary evolution has positioned Saudi Arabia as a significant contributor to global *Aspergillus* research. Despite foreign institutions contributing to approximately 20% of Saudi Arabian *Aspergillus* research, genuine international co-authorship remains low at 3.48%, revealing a gap between institutional presence and substantive intellectual collaboration. To address this, a multifaceted strategy is recommended, including the establishment of bilateral funding programs with co-principal investigator models, matched institutional contributions, and jointly developed proposals to ensure shared leadership. Incentivizing international co-authorship through recognition, financial rewards, and support for publication costs can further strengthen collaboration. Formalizing long-term partnerships via multi-year agreements, joint research centers, and dual faculty appointments, alongside structured monitoring of research output and collaboration quality, will enhance sustainability. Building interpersonal trust through regular communication, cultural exchange, and clear authorship and decision-making agreements is also essential. Saudi Arabia should leverage existing national science initiatives and expand regional partnerships to convert superficial foreign involvement into equitable, enduring research alliances that elevate the impact of its mycology research globally.

### 4.5 Taxonomic diversity and ecological representation

The exceptional diversity encompasses multiple significant taxonomic groups, underscoring the genus’s versatility in various ecological habitats across arid and semi-arid regions of Saudi Arabia. *A. flavus, A. parasiticus, and A. tamarii* highlight their agricultural importance, particularly with aflatoxin contamination in crops (Alotaibi et al., 2020; Awad et al., 2023). *A. niger, A. carbonarius*, and *A. brasiliensis* encompass both food rotting organisms and species of industrial significance, illustrating the dual function of this group in agriculture and biotechnology (Abdel-Azeem et al., 2016; Al-Dhabaan, 2021). *A. fumigatus* underscores the genus’s clinical significance, especially as the predominant etiological agent of invasive aspergillosis (Latge and Chamilos, 2019). The identification of uncommon and regionally relevant species, including *A. spelaeus, A. itaconicus*, and *A. egyptiacus*, indicates the necessity for comprehensive ecological investigation and potential biogeographical patterns that could enhance regional biodiversity and health risk evaluations. This extensive species documentation from prevalent, extensively researched taxa to specialized species demonstrates both research sophistication and comprehensive environmental monitoring. The identification of 108 species not only solidifies Saudi Arabia’s status as a biodiversity hotspot for *Aspergillus* but also establishes a strong basis for public health surveillance, agricultural management, and future research on the effects of climate and land use on fungal ecology.

## 5 Conclusion

Through the past five decades of *Aspergillus* species research in Saudi Arabia, the bibliometric study displays that the point is well-established and continues to grow gradually. Whereas this theme has acquired some attention in the scientific area, with 520 scientific articles published during 54 years with an annual growth rate of 5.24%. Further it has received 3,821 citations, which is an average of 7.35 per document. The field transitions from minimal activity in earlier years (pre-1990), to becoming established (1990–2005), to being consistently productive (2006–2012), to reaching a peak period of productivity (2013–2018) where it appears to have leveled off to a normal level of activity in recent years (2019–2024). The data are alarming in terms of concentration; for instance, 58% of the research outputs are from King Saud University and King Abdulaziz University. It also reflects in regional cooperation, which occurs in a hub-and-spoke model, with Saudi Arabia at the center, and Egypt acting as the main partner in all international cooperation. There are some driving themes that are already known from strategic mapping such as soil ecology and methods of fungus isolation, but also some are quite new and are very important but require more research, especially in the areas of molecular approaches and contaminant detection. The high observed taxonomic diversity of individuals collected, 108 different isolates. Which consider about 23.8% of all known species shows how rich the ecosystems are in Saudi Arabia. Further most of the articles has focused on main five species *A. niger, A. flavus, A. fumigatus, A. terreus*, and *A. ochraceus*. Also, citation analysis confirms that Saudi Arabian contributions are becoming more well-known. Whereas recent articles (2011–2020) makes up 40% of the most cited papers and indicates citation rates that are rising every year. This trend, along with the steady flow of research in recent years, implies that *Aspergillus* research in Saudi Arabia has grown from a period of rapid growth to a well-established, multidisciplinary research ecosystem that supports national goals in biotechnology, food security, and public health. To build on this foundation, national policy actions should be considered. These include the integration of fungal diagnostics into public hospital protocols to address clinical underdiagnosis, the use of molecular techniques such as metagenomics for environmental surveillance and antifungal resistance monitoring, and the establishment of a national *Aspergillus* biodiversity database. Furthermore, funding mechanisms and joint grant schemes should be developed to promote meaningful international collaborations beyond superficial co-authorship. Strengthening data-sharing platforms and cross-institutional capacity-building efforts will further position Saudi Arabia as a leader in regional mycological research. In forthcoming years, opportunities are anticipated to enhance international collaboration networks, refine methodological approaches with particular emphasis on molecular techniques, integrate specialized research areas with more generalized topics, extend environmental studies beyond soil investigations, and translate foundational knowledge into practical business applications.

## Data Availability

The original contributions presented in this study are included in this article/Supplementary material, further inquiries can be directed to the corresponding author.
