# Supplementary material for: Unveiling the distribution and research patterns of Aspergillus spp. in Saudi Arabia: a systematic and bibliometric analysis
Source: Front Microbiol. 2025 Aug 19;16:1638271. doi: 10.3389/fmicb.2025.1638271 (PMC12401964; doi:10.3389/fmicb.2025.1638271)
Supplement: Supplementary file 1 [file Table_1.docx]

Supporting data, Table 1 presents the remaining sources for the isolation of *Aspergillus* spp.

| General Source | Specific Source | Total Studies | Total Different Sources |
| --- | --- | --- | --- |
| Food | Food Samples | 39 | 32 |
| Food | Dried Friuts |  |  |
| Food | Pear fruit |  |  |
| Food | Potato chips |  |  |
| Food | White cheese |  |  |
| Food | Bread |  |  |
| Food | Juice Samples |  |  |
| Food | Honey |  |  |
| Food | Meat |  |  |
| Food | Spices |  |  |
| Food | Aqua Feed |  |  |
| Food | Noodles |  |  |
| Food | Food-industrial Wastes |  |  |
| Food | Jarish |  |  |
| Food | Qursan |  |  |
| Food | Fast Food |  |  |
| Food | Camel Feed |  |  |
| Food | Retail Table Eggs Shells |  |  |
| Food | Peach Peels |  |  |
| Food | Market Food |  |  |
| Food | Mushrooms Broth Cube |  |  |
| Food | Pasta |  |  |
| Food | Canned Meat |  |  |
| Food | Biscuits |  |  |
| Food | Poultry Feedstuffs |  |  |
| Food | Poultry Feedstuffs |  |  |
| Food | Buffalo Meat |  |  |
| Food | Cattle Meat |  |  |
| Food | Orange Peel |  |  |
| Food | Banana Peel |  |  |
| Food | Lemon Peel |  |  |
| Food | Capsicum |  |  |
| Industrial | Fabrics: Cotton | 17 | 15 |
| Industrial | Fabrics: Lenin |  |  |
| Industrial | Fabrics: Silk |  |  |
| Industrial | Fabrics: Wool |  |  |
| Industrial | Fabrics: Nylon |  |  |
| Industrial | Fabrics: Polyester |  |  |
| Industrial | Fabrics: Blended cotton (75%) and Polyester (25%) |  |  |
| Industrial | Fabrics: Blended cotton (65%) and Polyester (35%) |  |  |
| Industrial | Cosmetic Products |  |  |
| Industrial | Refrigerator and Freezer Doors |  |  |
| Industrial | Cell Phones |  |  |
| Industrial | Oily Sewage Dump |  |  |
| Industrial | Paper Currency |  |  |
| Industrial | Paper Money |  |  |
| Industrial | Student Lab Coats |  |  |
