# Supplementary material for: Unveiling the distribution and research patterns of Aspergillus spp. in Saudi Arabia: a systematic and bibliometric analysis
Source: Front Microbiol. 2025 Aug 19;16:1638271. doi: 10.3389/fmicb.2025.1638271 (PMC12401964; doi:10.3389/fmicb.2025.1638271)
Supplement: Supplementary file 3 [file Data_Sheet_1.docx]

Illustration for the five primary strains, isolated from KSA, are utilized in various research, demonstrating the distinct sources from whence they were derived.


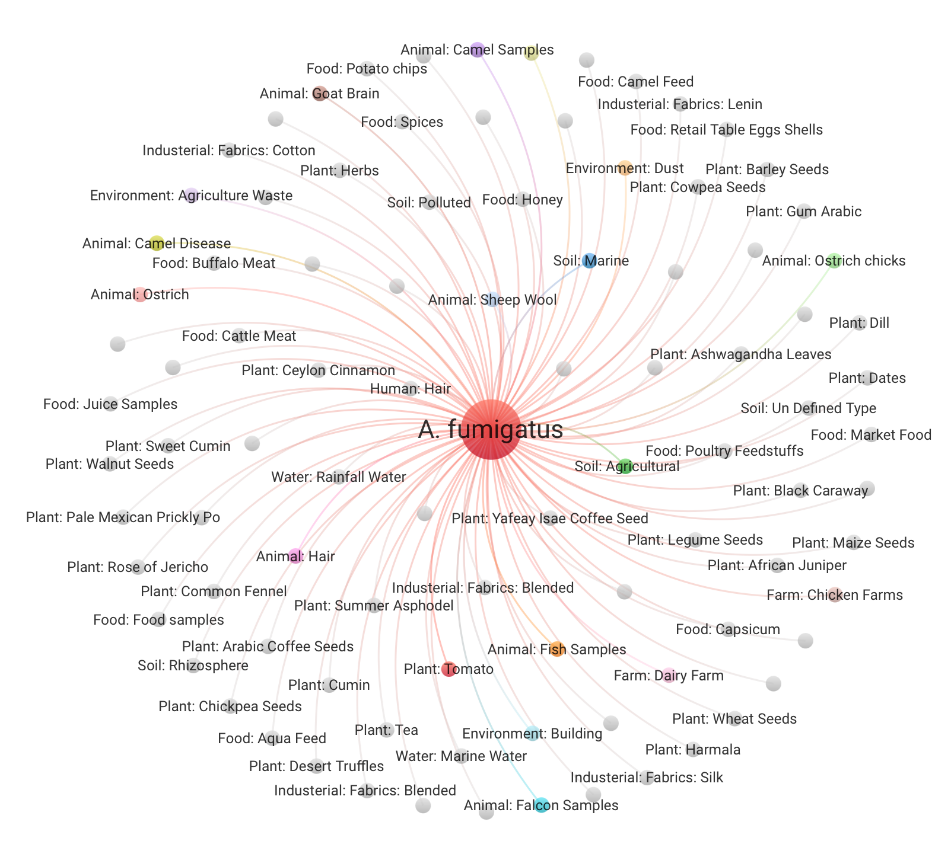


Figure 1


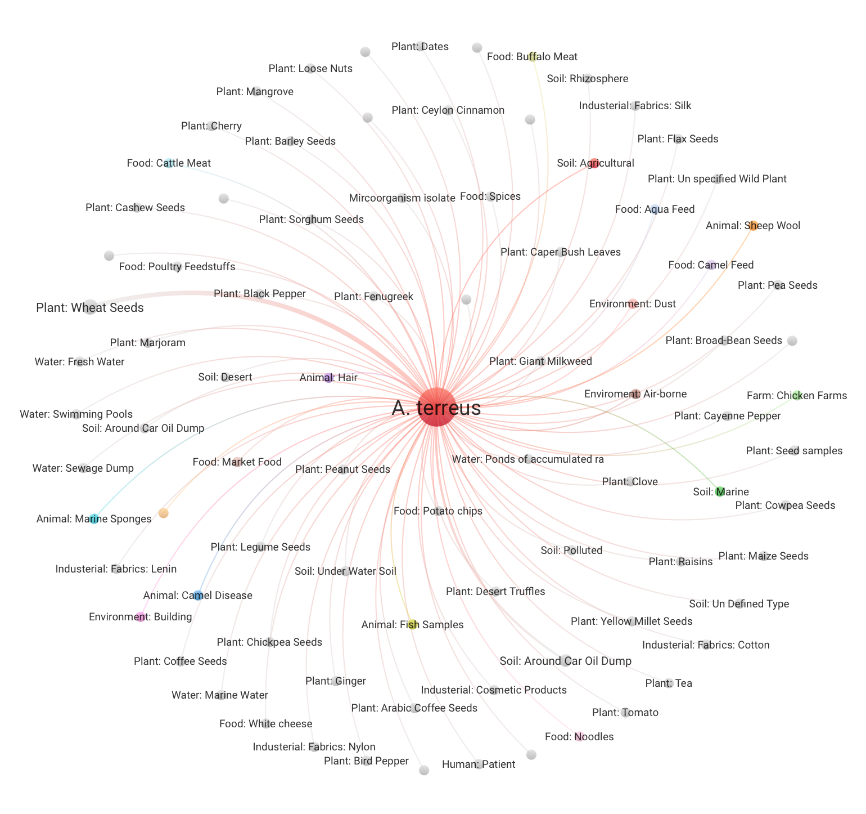


Figure 2


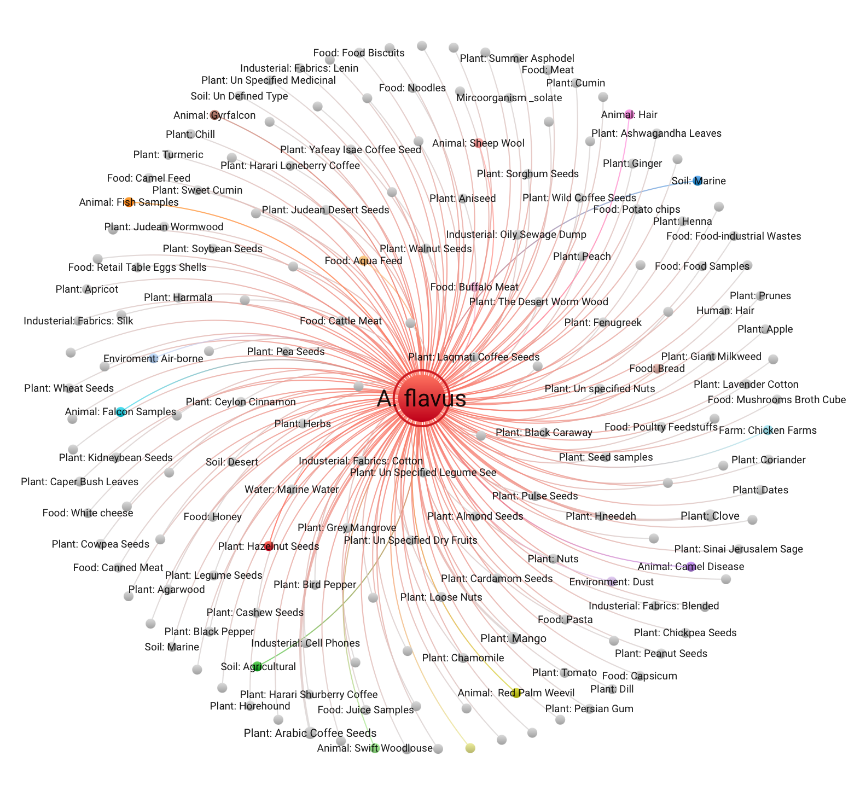


Figure 3


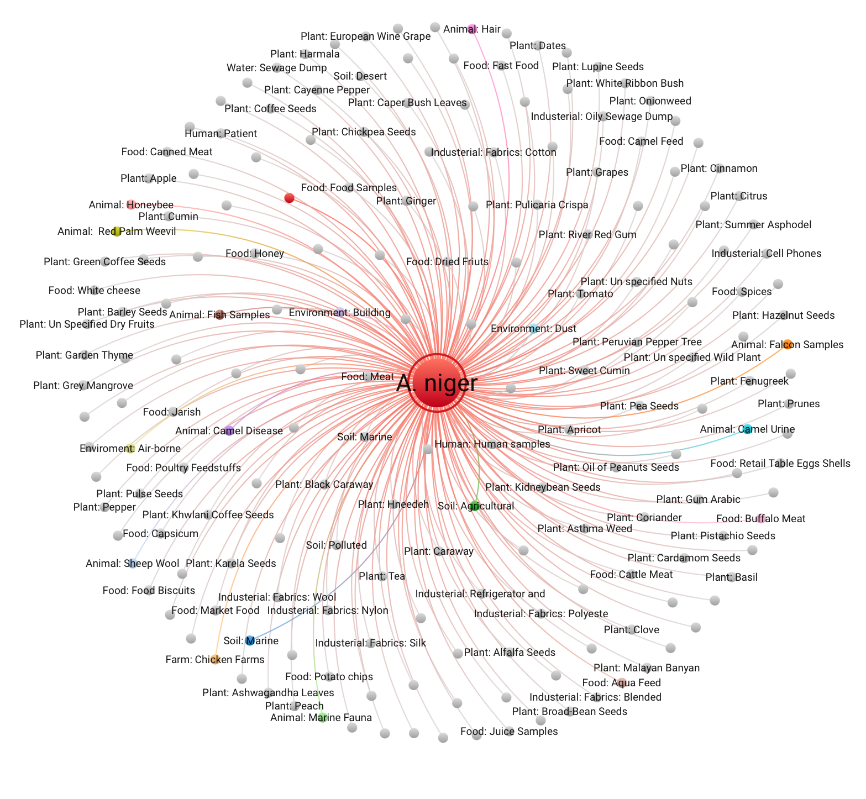


Figure 4


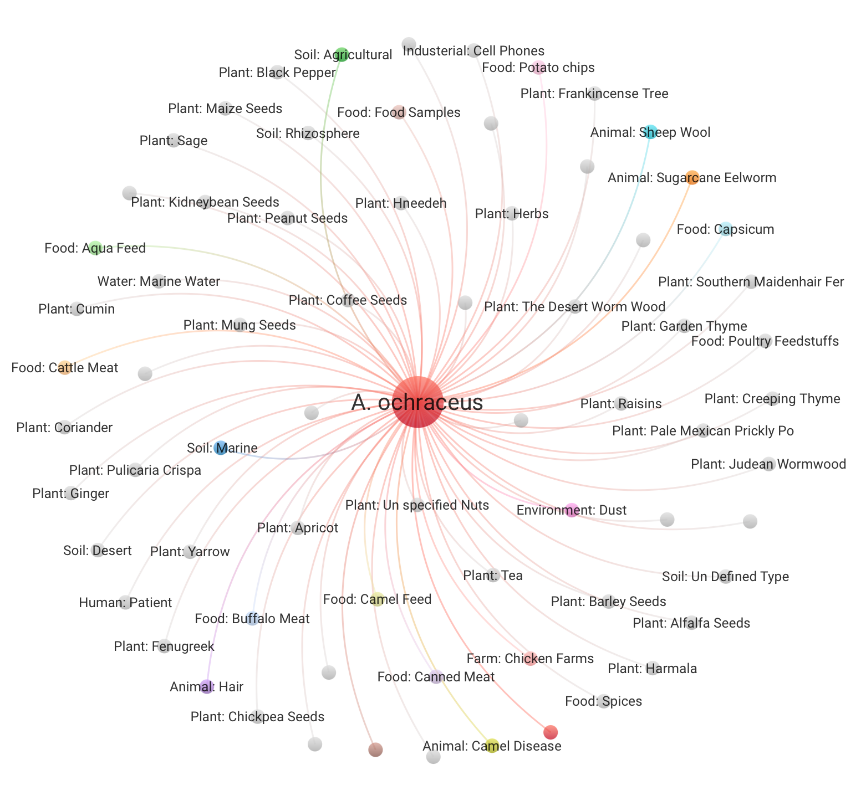


Figure 5
